# Ready-to-use 3D bioprinted scaffolds from natural materials loaded with patient’s PRGF for personalized skin regeneration

**DOI:** 10.1016/j.isci.2026.116185

**Published:** 2026-06-08

**Authors:** Lidia Maeso, Eduardo Anitua, Roberto Tierno, Mohammad Hamdan Alkhraisat, Edurne Alonso, Jon Luzuriaga, Jon Zarate, Felipe Goñi, Tatiane Eufrásio-da-Silva, Mohammadsadegh Nadimifar, Sadegh Ghorbani, Aziz Maleki, Alireza Dolatshahi-Pirouz, Gorka Orive

**Affiliations:** 1 Basque Sustainable Pharmacy and Biotherapy Research Group, School of Pharmacy, University of the Basque Country (UPV/EHU), Vitoria-Gasteiz, Spain; 2 Bioaraba Health Research Institute, 01009 Vitoria-Gasteiz, Spain; 3 Regenerative Medicine Laboratory, BTI-Biotechnology Institute, 01007 Vitoria-Gasteiz, Spain; 4 University Institute for Regenerative Medicine and Oral Implantology-UIRMI (UPV/EHU-Fundación Eduardo Anitua), Vitoria-Gasteiz 01007, Spain; 5 Department of Oral and Maxillofacial Surgery, Oral Medicine and Periodontology Faculty of Dentistry, University of Jordan, Amman 11942, Jordan; 6 Department of Cell Biology and Histology, Faculty of Pharmacy, University of the Basque Country (UPV/EHU), 01006 Vitoria-Gasteiz, Spain; 7 GAIKER Technology Centre, Basque Research and Technology Alliance (BRTA), Zamudio, Spain; 8 Department of Health Technology, Technical University of Denmark, 2800 Kongens Lyngby, Denmark; 9 Department of Materials Science and Engineering, Stanford University, Stanford CA, USA; 10 Zanjan Pharmaceutical Nanotechnology Research Center (ZPNRC), and Department of Pharmaceutical Nanotechnology, School of Pharmacy, Zanjan University of Medical Sciences, Zanjan 45139-56184, Iran

**Keywords:** Biological sciences

## Abstract

Chronic skin wounds demand individualized regenerative approaches that integrate structural support with controlled biological activity. To address this need, this study develops a ready-to-use (RTU) bioprinted scaffold generated from an extrusion-screened gelatin-alginate bioink selected for its rheological and mechanical behavior. The scaffold is ionically crosslinked, lyophilized, and rehydrated with autologous platelet-rich growth factors (PRGFs), yielding a mechanically stable matrix with favorable swelling, degradation, and viscoelastic properties. PRGF loading enables sustained release of bioactive factors for 48 h. *In vitro* assays with fibroblasts demonstrate cytocompatibility, adhesion, and proliferation, while *ex vivo* organotypic human skin cultures show reduced cellular damage and inflammatory markers together with enhanced extracellular matrix remodeling, evidenced by a gradual transition toward collagen type I and enhanced elastin deposition. These results highlight how combining bioprinting, acellular biomaterials, and autologous growth factor delivery can create customizable platforms for patient-specific wound therapy with translational potential for advanced clinical wound care.

## Highlights

• Ready-to-use 3D bioprinted scaffolds from gelatin and alginate
• On-demand personalization using autologous PRGF loading
• Bioink shows shear-thinning, self-healing, and enhanced mechanics upon crosslinking
• *Ex vivo* human skin model shows reduced inflammation and ECM remodeling

## Graphical abstract

**Figure:**
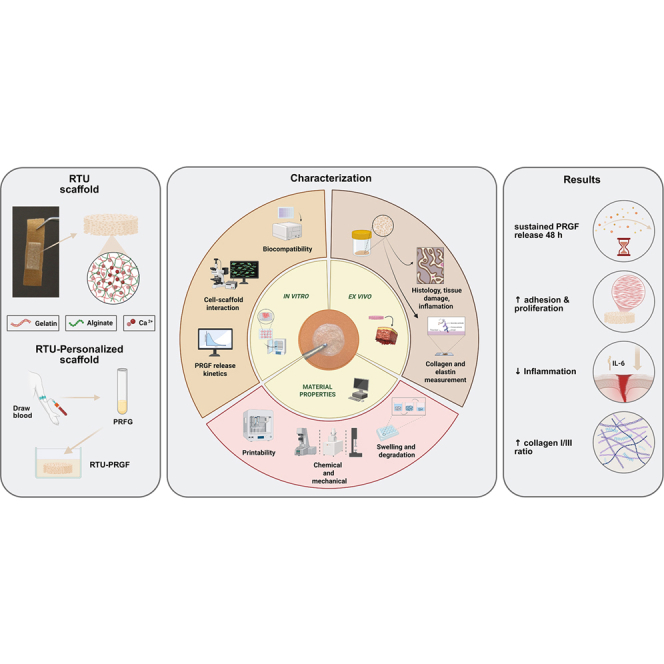

## Introduction

Skin wounds represent a major healthcare challenge worldwide, with significant socio-economic and clinical implications, especially in the form of burns and chronic wounds (such as diabetic ulcers, pressure ulcers, or venous leg ulcers).^1^ These types of wounds are classified as chronic when they fail to progress through the typical stages of healing within a time frame of 3 months.^2^ Affecting millions of individuals annually, chronic wounds are associated with high morbidity, reduced quality of life, and substantial healthcare costs.^3^ According to the most recent estimates published in 2025, approximately 10.5 million people are affected by wounds each year in the United States, and in 2022 the associated healthcare costs were estimated at $148.65 billion.^4^^,^^5^ Additional costs may arise from complications such as infections, tissue necrosis, and amputations or the need for prolonged medical care. Chronic wounds result from a complex interplay between intrinsic factors such as age, underlying medical conditions, and genetic predisposition and extrinsic factors including pressure, shear, friction, and moisture. They are characterized by a prolonged inflammatory phase and a degradation of the extracellular matrix (ECM), which is essential for providing structural support to the skin tissue.^6^

The difficulty in treating chronic wounds often stems from other conditions such as diabetes, poor circulation, and immune system deficiencies, impairing the body’s natural healing mechanisms. In these cases, the wound healing process is disrupted at multiple levels, from cell migration to angiogenesis, necessitating the development of advanced regenerative therapies that can accelerate wound healing while minimizing complications.^4^^,^^7^^,^^8^ In recent years, research into bioactive materials and tissue engineering has provided new possibilities for treating these complex cases.^9^^,^^10^ Tissue engineering aims to address this global issue by integrating materials science, engineering, and biology.^11^ Specifically, skin tissue engineering focuses on developing scaffolds, hydrogels, and other biomaterials that can serve as templates to facilitate the repair of damaged skin tissue.^12^

Lately, significant advancements in tissue engineering have been achieved, particularly through the development of technologies such as 3D bioprinting, freeze-drying, and nanoengineering, as well as the incorporation of bioactive materials.^13^^,^^14^^,^^15^^,^^16^ Among these, 3D bioprinting has emerged as an advanced additive manufacturing technology that allows for the precise layer-by-layer deposition of cells, biomaterials, and growth factors, enabling the creation of complex tissue-like structures. This technology has revolutionized tissue engineering by allowing the fabrication of customizable scaffolds that mimic the structural and functional properties of natural tissues.^17^ Extrusion-based 3D bioprinting is the most widely used technique. It allows for precise control over the scaffold’s architecture, including porosity and layer thickness that are key features for ensuring adequate nutrient diffusion and cell infiltration.^18^ Moreover, the printed constructs can be tailored to match the exact geometry of a wound, improving their integration with native tissue and reducing the risk of postoperative complications such as infection or excessive scarring.^2^ Gelatin and alginate are among the most widely employed biomaterials in 3D bioprinting. Gelatin-alginate scaffolds are frequently used in tissue engineering due to their biocompatibility, biodegradability, and ability to support cell attachment and proliferation. Gelatin provides a favorable microenvironment for cell growth and function,^13^ whereas alginate contributes to the mechanical integrity and structural stability of the printed constructs.^19^ Importantly, both gelatin and alginate are naturally derived biomaterials with established clinical use in a range of topical medical devices, including wound dressings and absorbable hemostatic sponges. Their safety has been recognized by the US Food and Drug Administration (FDA), which classifies them as Generally Recognized as Safe (GRAS).^13^ Gelatin, for example, is recognized for its use as a medical-grade biomaterial in pharmaceuticals and wound dressings, and it is a main component in FDA-approved absorbable hemostatic sponges (such as GELFOAM).^20^^,^^21^^,^^22^ Similarly, alginate is incorporated into products intended for wound healing applications, and alginate-based dressings are cleared by the FDA as medical devices for wound management (e.g., LUOFUCON, COLLASORB, and DERMAPHYLYX).^23^^,^^24^^,^^25^ These regulatory approvals underscore the safety, biocompatibility, and compliance of both materials with strict FDA standards regarding toxicity, purity, and suitability for direct contact with human tissues. Therefore, scaffolds based on these materials have been suggested to combine biocompatibility, porosity, controlled biodegradability, and other favorable features, which may help mimic aspects of the skin microenvironment and support regeneration, vascularization, and infection control during wound healing.^26^^,^^27^

Ready-to-use (RTU) 3D bioprinted gelatin-alginate scaffolds integrated with autologous plasma rich in growth factors (PRGFs) present a promising approach for personalized therapy to enhance skin regeneration. PRGFs are easily obtained from the patient’s blood; furthermore, they contain a concentrated mixture of growth factors, including platelet-derived growth factor (PDGF), transforming growth factor β (TGF-β), vascular endothelial growth factor (VEGF), epidermal growth factor (EGF), basic fibroblast growth factor (FGFb), and insulin-like growth factor 1 (IGF-1), among others.^28^^,^^29^ These growth factors play essential roles in stimulating cellular proliferation, promoting angiogenesis, and enhancing collagen synthesis, suggesting that PRGFs may serve as a promising candidate for supporting skin regeneration.^30^ Such scaffolds can offer mechanical and milieu support to the wound site, and the incorporation of PRGFs creates a synergetic effect delivering a sustained release of growth factors to stimulate cellular activity and accelerate the healing process.

Preclinical studies and clinical case series have demonstrated that PRGF-based formulations significantly improve wound closure and contribute to superior tissue regeneration and restoration of skin function and structure compared to traditional treatments.^31^^,^^32^^,^^33^ The development of these innovative scaffolding systems offers promising opportunities for improving wound healing, particularly in chronic and hard-to-heal cases. In this context, we introduce a new generation of RTU, medical-grade scaffolds enriched with PRGFs (RTU-PRGF). The RTU approach allows for immediate application without the need for additional in-lab preparation or post-processing, representing a significant advance over conventional, labor-intensive therapies.^34^^,^^35^^,^^36^^,^^37^^,^^38^^,^^39^ To achieve this, first the RTU gelatin-alginate 3D bioprinted scaffolds were fabricated and thoroughly characterized in terms of swelling, degradation, chemical, mechanical, and rheological properties. To achieve patient-specific relevance through personalization, PRGF was incorporated into the RTU scaffolds, and fibroblast cells (human dermal fibroblasts [HDFs] and L-929) were cultured on these RTU-PRGF scaffolds to evaluate their biological potential. Finally, *ex vivo* studies demonstrated the capacity of these systems to regenerate skin injuries. The RTU format of these scaffolds can markedly enhance clinical translation by enabling straightforwardness of handling, storage, and personalized application at the point of healthcare.

## Results and discussion

**Figure 1:**
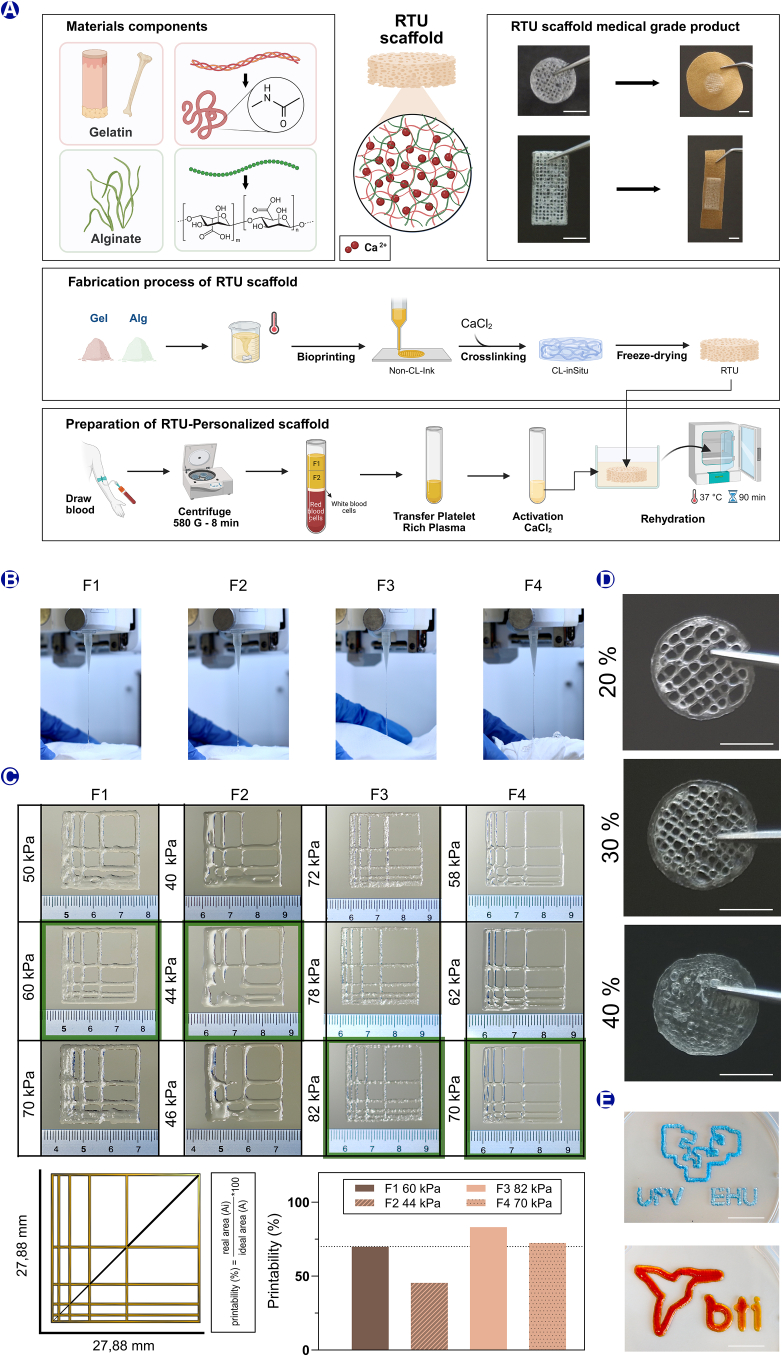
Overview of the development, fabrication, and printability assessment of RTU and RTU-personalized bioprinted scaffolds (A) Schematic representation of the RTU scaffold composition and stepwise fabrication process of both RTU and RTU-personalized systems, together with macroscopic images of the medical-grade RTU scaffold product (scale bars, 5 mm). Printing test of gelatin-alginate hydrogel bioinks: (B) filament formation test of four gelatin-alginate bioinks (F1, F2, F3, and F4), showing that all formulations produced continuous filaments upon extrusion, confirming their suitability for 3D bioprinting. (C) Comparison between the reference 3D-designed model and the optical images of the bioprinted constructs generated with each bioink at different extrusion pressures, to assess the effect of pressure on print fidelity. A graph summarizes the printability performance of the four bioinks. (D) Printability at varying infill densities. Optical images of F3-based hydrogels printed with 20%, 30%, and 40% infill, highlighting structural differences associated with infill variation (scale bars, 5 mm). (E) Evaluation of complex structure printability. The selected formulation (F3), stained with methylene blue and alizarin, was assessed for its ability to reproduce complex geometries at 30% infill. Optical images confirm the capability of F3 to generate intricate scaffold structures (scale bars, 20 mm).

RTU-personalized scaffolds were designed for skin injury treatment using two sustainable, FDA-approved natural materials. Gelatin-alginate scaffolds were fabricated via 3D bioprinting, following optimization of bioink formulations, and subsequently underwent crosslinking, lyophilization, and rehydration with PRGFs, as illustrated in Figure 1A. Initially, bioinks were prepared by combining gelatin and alginate in varying ratios through a simple mixing protocol at 60°C. Four formulations (F1–F4) were selected for 3D printing using a CELLINK BIO X bioprinter, yielding scaffolds with distinct and well-defined geometries. After lyophilization, the printed hydrogels transitioned from a fully translucent gel to an opaque and more rigid structure, resulting in an easy-to-handle dehydrated product with reduced risk of microbial contamination. These scaffolds showed optimal potential for clinical application, particularly as a main component of an adhesive wound dressing, due to their bioactivity, storability, and straightforward application. The physicochemical properties of the bioinks and scaffolds were first characterized, followed by biological and medical performances evaluations. RTU-personalized scaffolds were obtained by rehydrating the lyophilized RTU scaffold with PRGFs through a straightforward loading protocol, facilitating subsequent *in vitro* and *ex vivo* wound-healing assessments.

### Printability

Printability is a key parameter in the development of bioinks for extrusion-based 3D bioprinting, as it directly influences the geometric fidelity, structural integrity, and reproducibility of the printed constructs.^40^ In this study, printability was assessed both qualitatively and quantitatively.

Among all tested formulations, four candidate bioinks (F1–F4) were selected for their capacity to form continuous, homogeneous filaments and retain well-defined shapes upon extrusion, as demonstrated by the qualitative filament formation test (Figure 1B). Subsequently, a quantitative analysis was performed by comparing the printed area (Ai) to the ideal design area (A), as shown in Figure 1C. All formulations were printed using a CELLINK BIO X bioprinter under the same printing conditions (speed, nozzle size, and printbed temperature), with the exception of extrusion pressure. This parameter was adjusted individually for each ink to ensure optimal filament formation based on its rheological properties. The best-performing pneumatic extrusion pressure for each ink was selected for comparative analysis. The results revealed distinct differences in printing fidelity among the formulations. Bioink F3, which required the highest pressure to extrude (82 kPa), exhibited the highest printability value, approaching 80%, and showed high shape fidelity after printing. In contrast, bioink F2, extruded at the lowest pressure (44 kPa), showed poor structural definition, with a printability below 50%. Bioinks F1 and F4 yielded intermediate results, with printability values between 60% and 70%. These findings highlight the importance of achieving a balanced viscoelastic profile: overly viscous bioinks may cause clogging or require excessive pressure, while low-viscosity inks tend to spread or collapse after deposition. Considering both qualitative and quantitative results, bioink F3 was identified as the most suitable candidate for further physicochemical characterization and biological evaluation.

Additionally, bioink F3 was printed using a grid pattern with different infill densities ranging from 20% to 40% (Figure 1D). A 30% infill was selected, as it provided the best compromise between mechanical stability and porosity, offering sufficient structural rigidity while maintaining an open architecture suitable for cell infiltration and nutrient diffusion. To assess the ability of the optimized bioink to accurately reproduce complex shapes, a key requirement for generating personalized scaffolds tailored to the specific geometry of a wound, Bioink F3 was stained with methylene blue and alizarin solely for visualization purposes. The addition of these colorants did not alter the rheological behavior of the bioink, which extruded identically to the uncolored formulation. Using the stained bioinks, the logos of two collaborating institutions were successfully printed with high precision (Figure 1E), demonstrating the formulation’s resolution and customization potential.

### Rheological and compression mechanical characterization

**Figure 2:**
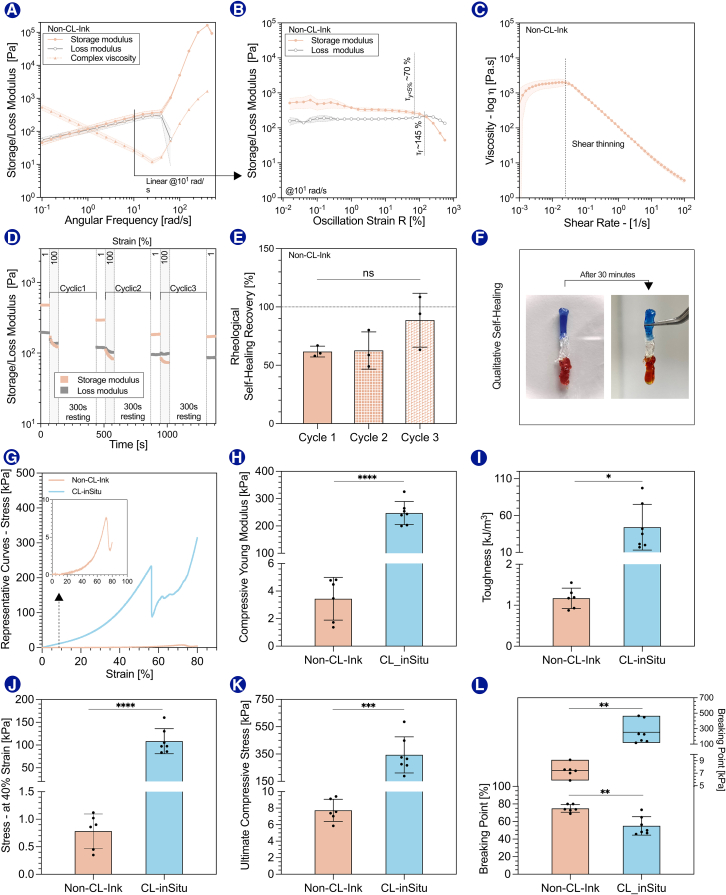
Comprehensive rheological and mechanical characterization of the bioprinting ink before and after ionic crosslinking Rheological characterization of the bioink, including dynamic frequency sweep (A), amplitude sweep (B) (storage modulus G′ and loss modulus G″ vs. shear strain), viscosity (C), self-healing capability (D), self-healing recovery across cycles (E), and a macroscopic image illustrating the self-healing behavior of the non-crosslinked ink (F). Mechanical characterization of the bioprinting ink before and after crosslinking with Ca^2+^ ions: compressive stress-strain curve (G), Young’s modulus (H), toughness (I), stress at 40% strain (J), ultimate stress (K), and breaking point (L) of the non-crosslinked (Non-CL-Ink) and *in situ* crosslinked (CL-inSitu) ink. Statistical significance is indicated as follows: not significant (ns); ∗*p* < 0.05, ∗∗*p* < 0.01, ∗∗∗*p* < 0.001, and ∗∗∗∗*p* < 0.0001.

To complement the printability study and better understand the physical behavior of the selected formulation, the rheological properties of bioink F3 in its non-crosslinked state were thoroughly characterized (Figure 2). Rheological performance is essential for extrusion-based bioprinting, as it governs both the ink’s behavior under shear and its capacity to recover post-deposition.^41^ The frequency sweep analysis showed that bioink F3 exhibited predominantly solid-like behavior, with the storage modulus (G′) consistently higher than the loss modulus (G″) as the angular frequency increased (0.8–100 rad/s) (Figure 2A). This viscoelastic profile confirms a stable polymer network capable of maintaining structural fidelity after extrusion. The complex viscosity values aligned with the trend observed in printability, indicating that the bioink could sustain its shape post-printing. Amplitude sweep analysis further revealed that G′ remained dominant up to approximately 70% strain, defining the linear viscoelastic region (LVR) of the bioink (Figure 2B). Beyond this point, both G′ and G″ began to decrease, indicating the disruption of internal structure under higher deformation—a critical feature for ensuring responsiveness during extrusion. The flow curve confirmed the ink’s shear-thinning behavior, characterized by a rapid decrease in viscosity with increasing shear rate (Figure 2C). This pseudoplastic nature is advantageous for extrusion printing, allowing smooth flow through the nozzle under pressure while promoting rapid gel recovery upon deposition, thus preserving the printed geometry.^42^

To evaluate the self-healing capacity of the ink, a time-sweep test was performed by alternating between high (100%) and low (1%) strain cycles (Figure 2D). After each cycle, the bioink showed partial but progressive recovery of its storage modulus, indicating the reformation of its internal network. The percentage of rheological recovery is quantified in Figure 2E, with values improving across three consecutive cycles, suggesting cumulative adaptation and resilience of the material under repeated mechanical stress. Finally, to visually demonstrate the self-healing capacity of the ink, bioink F3 was dyed with methylene blue and alizarin red, and three adjacent filaments were manually extruded in close contact. As shown in Figure 2F, after 30 min of incubation, the filaments spontaneously fused into a single, continuous structure composed of three distinct color segments. The integrated construct could be lifted from one end using forceps without breaking, confirming the ink’s ability to self-repair and form cohesive networks post-deposition. Importantly, this work provides one of the first systematic rheological and visual demonstrations of self-healing in a simple gelatin-alginate bioink specifically tailored for bioprinting and translational wound healing applications. By quantifying cyclic recovery and illustrating filament fusion, we highlight not only the material’s resilience under repeated mechanical stress but also its functional relevance for maintaining structural integrity during extrusion, handling, and *in situ* wound deployment. This combination of quantitative and qualitative analysis establishes a framework for evaluating self-healing in clinically oriented bioinks and distinguishes our study from prior reports on gelatin-alginate systems.^43^^,^^44^^,^^45^^,^^46^ Overall, these rheological results confirm that bioink F3 possesses the ideal combination of elastic behavior, shear-thinning flow, and self-healing capacity required for high-performance 3D bioprinting to create complex geometries.^42^

Additionally, biomechanical properties are key factors in tissue engineering, particularly in skin regeneration, as native skin exhibits notable elasticity and mechanical resilience.^27^ To assess whether the engineered hydrogels were mechanically suitable for skin applications, uniaxial compression tests were performed to determine their elastic Young modulus, toughness (calculated as the area under the stress-strain curve), stress at 40% strain, breaking point (both in kPa and %), and ultimate compressive stress (i.e., the maximum stress the scaffold can withstand before failure).

Comparative analysis between the non-crosslinked ink (Non-CL-ink) and the *in situ* crosslinked ink version (CL-inSitu) revealed a marked enhancement in biomechanical performance upon crosslinking (Figures 2G–2l). The CL-inSitu group exhibited significantly higher compressive strength, elastic modulus, and toughness, with values approximately 100 times greater than those of the non-crosslinked group. Specifically, the elastic modulus increased from ∼3 to ∼250 kPa (Figure 2H), the toughness value from ∼1 to ∼45 kJ/m^3^ (Figure 2I), and the ultimate compressive stress rose from ∼8 to ∼350 kPa (Figure 2K). Although these values are somewhat lower than those typically reported for native human skin, which typically exhibits an elastic modulus between 60 and 850 kPa,^47^^,^^48^ they still fall within the range considered suitable for engineered skin substitutes. Indeed, while fibroblasts can survive on substrates with a wide range of stiffness, previous studies have shown that elastic moduli around 200 kPa, comparable to the values obtained here, can enhance fibroblast proliferation, migration, and ECM deposition, key processes in skin repair and remodeling, compared to softer substrates (∼16–20 kPa).^49^^,^^50^^,^^51^ Moreover, the mechanical stability observed in CL-inSitu scaffolds suggests that they could maintain integrity during handling and implantation, while still offering a compliant environment mimicking the biomechanical cues of native skin tissue.

### Determination of microstructure and elemental composition

**Figure 3:**
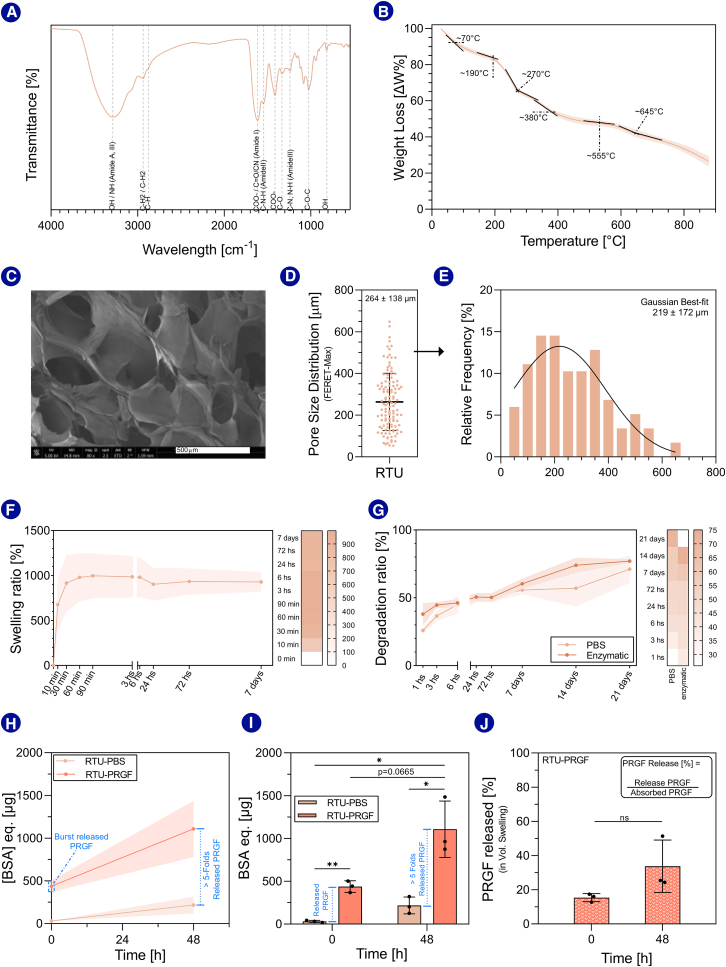
Physicochemical characterization and growth factor release of RTU scaffolds. Chemical characterization of the hydrogel, including (A) FTIR spectroscopy and (B) TGA. (C) SEM image of the lyophilized RTU scaffold highlighting its porosity and corresponding (D) pore size distribution and (E) frequency. (F) Swelling behavior and (G) degradation profile of the RTU scaffold under physiological conditions (PBS) and enzymatic degradation. Evaluation of protein release after 48 h: (H) release of BSA (μg) over 48 h, shown as a time-dependent curve, (I) comparison of released BSA content at 0 and 48 h and (J) released PRGF expressed as a percentage of the initially adsorbed amount, demonstrating the RTU scaffold’s capacity to retain and gradually deliver bioactive molecules, after rehydration. Statistical significance is indicated as follows: not significant (ns); ∗*p* < 0.05, ∗∗*p* < 0.01, ∗∗∗*p* < 0.001, and ∗∗∗∗*p* < 0.0001.

The chemical interactions within the gelatin-alginate network, as well as those formed during crosslinking with Ca^2+^ ions, were assessed by Fourier transform infrared spectroscopy (FTIR) and thermogravimetric analysis (TGA). FTIR spectra revealed characteristic peaks for alginate, including a broad absorption around 3,232 cm^−1^ corresponding to hydroxyl (–OH) groups, and a peak at 2,917 cm^−1^ attributed to CH_2_ stretching. Peaks associated with asymmetric and symmetric stretching of carboxylate groups (COO^−^) appeared at 1,598 cm^−1^ and 1,403 cm^−1^, respectively, corresponding to the typical signatures of polysaccharide-based alginate materials. In addition, signals corresponding to glycosidic bonds were observed at 1,304 cm^−1^ (C–O stretching) and 1,010 cm^−1^ (C–O–C asymmetric stretching). The scaffolds also exhibited characteristic gelatin bands. These included the broad band at ∼3,232 cm^−1^ (overlapping OH and NH stretching, corresponding to Amide A and Amide III), a peak at 2,917 cm^−1^ for CH_2_ stretching, and a prominent absorption at 1,598 cm^−1^, associated with C=O stretching and C–N bending (Amide I). Additional gelatin-associated peaks were detected at 2,862 cm^−1^ (CH stretching), 1,532 cm^−1^ (C–N–H bending, Amide II), 1,228 cm^−1^ (C–N stretching and N–H bending, Amide III), and 804 cm^−1^ (OH group), confirming the successful integration of gelatin into the scaffold structure (Figure 3A).^52^^,^^53^ These results are summarized in Table S1 in the Supporting Information.

The thermal stability of the lyophilized RTU scaffolds from F3 was evaluated using TGA to investigate their compositional profile and thermal resistance, which are critical parameters for sterilization, storage, and biomedical application.^54^ As shown in Figure 3B, the TGA curve revealed a multistep degradation profile, indicating the presence of different thermolabile components within the scaffold structure.

The first noticeable weight loss occurred around 70°C, mainly corresponding to the evaporation of residual moisture and loosely bound water. A second degradation event was observed at approximately 190°C–270°C, likely associated with the thermal decomposition of low-molecular-weight gelatin and alginate chains, including the rupture of glycosidic bonds and partial protein denaturation.^52^^,^^53^ A more substantial weight loss occurred between 380°C and 555°C, attributed to the breakdown of the main polymeric backbone and organic matrix carbonization.^55^ A final minor degradation phase was identified near 645°C, suggesting the presence of thermally stable remnants or char formation. These well-defined degradation stages confirm the structural complexity of the RTU scaffold and its adequate thermal resistance under physiologically relevant and processing conditions. Overall, the RTU gelatin-alginate-based scaffolds exhibited thermal stability up to ∼190°C, well above the temperatures typically used in common sterilization methods such as autoclaving (121°C) or dry heat (160°C–170°C).^56^ This ensures that the scaffolds can withstand standard handling, storage, and sterilization procedures without significant degradation of their polymeric structure, thereby supporting their practical applicability for clinical use in regenerative medicine.

### Porosity

Microstructural characterization and pore size analysis of the lyophilized RTU scaffolds were performed using scanning electron microscopy (SEM). Although bulk porosity quantification could provide complementary structural information, the primary objective of this study was to evaluate pore morphology and functional performance. In the context of skin tissue engineering, scaffold porosity plays a pivotal role—not only in facilitating cell infiltration, nutrient diffusion, and oxygen transport but also in modulating the retention and controlled release of bioactive molecules such as those present in PRGFs.^57^ SEM analysis revealed that the RTU scaffolds exhibited a highly porous and interconnected architecture (Figure 3C), a key feature for supporting cell migration, attachment, and tissue remodeling.^58^ As shown in Figures 3D and 3E, the RTU scaffolds exhibited an average pore diameter of 264 ± 138 μm, with the majority of pores falling within the 100–400 μm range, as indicated by the relative frequency distribution. This pore size range is suitable for dermal tissue regeneration, as pores larger than 100 μm allow fibroblast migration without compromising dermal formation, and even larger pores (over 200 μm) further promote neovascularization, nutrient exchange, and ECM deposition.^59^^,^^60^ Importantly, unlike conventional sponge-based scaffolds, the 3D-printed RTU scaffolds preserve a predefined and reproducible architecture after lyophilization and rehydration. SEM analysis confirmed that the macroscopic geometry, filament deposition, and interconnected pore network established during printing remained discernible, despite minor microstructural relaxation commonly observed in soft hydrogel systems.^61^ This controlled architecture not only supports cell infiltration and nutrient diffusion but also provides a structurally predictable environment for adsorption and release of bioactive molecules, highlighting the novelty and functional advantage of the RTU system over traditional sponge-like scaffolds.

Notably, the scaffold porosity also influenced the behavior of PRGFs within the system. High porosity and interconnected pore network of the RTU scaffolds likely facilitated rapid fluid uptake during rehydration, as described in Section 3.5, promoting the effective adsorption of PRGF-derived proteins onto the inner surfaces of the RTU scaffold. This open architecture supports the initial burst release of growth factors, as further studied in Section 3.6, which can be advantageous in the early stages of wound healing by rapidly activating regenerative processes. However, it may also accelerate the release kinetics, potentially limiting sustained exposure over longer periods. This trade-off is particularly relevant in skin regeneration, where a prolonged and localized presence of factors such as PDGF, VEGF, and IGF-1 has been shown to enhance tissue repair and remodeling.^62^^,^^63^

### Swelling ability and degradation profile

The swelling properties of the lyophilized RTU scaffolds were evaluated using the previously described equation (Equation 1). Wettability is a key factor in ensuring effective absorption of body fluids and nutrients, as well as facilitating metabolite exchange.^64^^,^^65^ Moreover, the swelling capacity provides insights into the interaction between the scaffold and its surrounding environment; however, it must be carefully controlled to avoid excessive degradation.^66^^,^^67^ In this context, the swelling behavior was particularly relevant to determine the optimal incubation time for the rehydration process (PBS or PRGFs), ensuring maximum fluid absorption. Results showed that swelling reached its peak after 90 min of rehydration, which was subsequently established as the standard incubation time for RTU-based scaffold rehydration in all further experiments (Figure 3F).

A temporary scaffold that supports tissue ingrowth while undergoing controlled degradation in synchrony with tissue regeneration remains a critical requirement, particularly in skin tissue engineering, where rapid but orderly regeneration of epidermal and dermal layers is essential. Therefore, biodegradability is considered one of the most important features for scaffolds intended for tissue regeneration.^68^^,^^69^

The bioprinted lyophilized RTU scaffolds remained structurally stable for the first 72 h under hydrolytic conditions (PBS), with degradation exceeding 50% thereafter (Figure 3G). This degradation pattern aligns with previous observations suggesting that the degree of crosslinking significantly influences the structural integrity and mechanical performance of hydrogels, as demonstrated by Pan et al.^70^ Based on these findings, RTU-PRGF scaffolds were replaced every 48 h in *ex vivo* applications.

To better approximate the enzymatic environment of healing wounds, additional degradation studies were conducted under collagenase-containing conditions. This experiment was designed following previously reported approaches describing the role of matrix metalloproteinases (MMPs) in the degradation of extracellular-matrix-based biomaterials.^71^ MMPs, including collagenases and gelatinases such as MMP-1, MMP-2, MMP-8, and MMP-9, are key enzymes responsible for collagen and elastin degradation during wound healing. Therefore, incorporating an enzymatic environment representative of *in vivo* remodeling processes was considered essential to more accurately evaluate scaffold behavior. A crude collagenase formulation (collagenase type I, Sigma #C0130), containing a mixture of collagenases and associated proteases, was selected rather than a purified single enzyme. This approach better reflects the cooperative proteolytic activity occurring during ECM remodeling *in vivo* and is widely adopted for assessing the degradation of collagen-based biomaterials under physiologically relevant conditions.

Under enzymatic conditions, the degradation profile followed the same overall trend observed in PBS, although with accelerated mass loss. Approximately 50% degradation was reached within the first 24 h, increasing progressively to nearly 77% after 3 weeks. These findings confirm that the scaffold undergoes controlled yet responsive degradation when exposed to proteolytic activity, supporting its suitability for dynamic wound environments where enzymatic remodeling plays a central role.

### Growth factor release profile

Prior to evaluating the release kinetics, the PRGF loading efficiency was quantified to determine the proportion of bioactive material effectively retained within the scaffold after rehydration. Lyophilized scaffolds (initial dry weight: 5.63 ± 2.18 mg) were rehydrated with 200 μL of PRGFs and allowed to swell for 90 min. After rehydration, the scaffolds reached a final weight of 57.88 ± 12.76 mg, corresponding to a net weight increase of 52.25 mg. Assuming a specific density of 1.0084 g/mL for PRGFs, this mass gain corresponds to an absorbed volume of 52.69 μL. Based on these values, the PRGF loading efficiency was calculated as 26.35%, indicating that approximately one-quarter of the initially applied PRGFs was effectively retained within the scaffold matrix.

Following loading, the growth factor liberation profile was further evaluated through a total protein quantification using the BCA assay (Figure 3H). The comparison of released BSA content at 0 and 48 h between RTU-PBS (rehydrated in PBS) and RTU-PRGF scaffolds (Figure 3I) revealed a marked 5-fold increase in PRGF release at 48 h post-loading by rehydration, with approximately 30% of the initially adsorbed protein content being liberated (Figure 3J). These findings suggest that while a significant portion of the PRGFs is efficiently released during the early phase, a fraction remains retained within the scaffold matrix, likely through physical entrapment or surface interactions. This retained reservoir may serve as a secondary source of growth factors, gradually released as the RTU-PRGF scaffold undergoes degradation. Therefore, controlling the scaffold porosity is essential not only for enabling proper cell-scaffold interactions but also for optimizing the delivery profile of therapeutic agents. Future work could involve adjusting the initial PRGF volume applied, thereby reducing excess unretained fraction and improving overall cost-effectiveness and fine-tuning the balance between porosity and retention capacity to achieve a more controlled release of PRGF components, ultimately enhancing their performance in wound healing applications. Nevertheless, given the importance of preventing infection and maintaining hydration to support tissue regeneration through regular wound dressing renewal,^72^^,^^73^ the use of fresh RTU-PRGF scaffolds in a clinical setting would ensure continuous exposure of the skin injury to therapeutic factors.

From a translational perspective, the feasibility of PRGF-based personalization was also considered. A single minimally invasive blood draw (e.g., one standard 9 mL collection tube) typically yields approximately 2 mL of active PRGFs. Given that less than 200 μL are required to rehydrate and functionalize a 1 cm diameter scaffold, the amount obtained from a routine blood extraction would be sufficient to prepare scaffolds of clinically relevant dimensions, including coverage of wounds approximating the median chronic wound area (∼4 cm^2^), or even multiple scaffolds if necessary.^74^ While patient-specific variability in PRGF yield may exist, these estimates support the practical applicability of this personalized strategy within standard clinical workflows. By combining 3D bioprinting with autologous PRGF functionalization, the RTU scaffolds achieve a level of spatial and biological control unattainable with conventional gelatin-alginate dressings. This strategy creates a personalized, biologically active platform that delivers patient-derived growth factors in a controlled and sustained manner, highlighting both its functional novelty and translational potential.

### Biological performance

#### Cell viability and biocompatibility study

A fundamental aspect in tissue engineering construct development is the assessment of cytocompatibility.^75^ In this study, the biocompatibility of the RTU gelatin-alginate-based scaffolds (rehydrated in PBS; RTU-PBS) and RTU-PRGF scaffolds was assessed through both qualitative and quantitative approaches using L-929 fibroblasts. Two complementary assays were performed: (1) a Live/Dead staining assay to visualize cell viability over time and (2) cytotoxicity assays following the ISO 10993 standard for biological evaluation of medical devices.^76^

**Figure 4:**
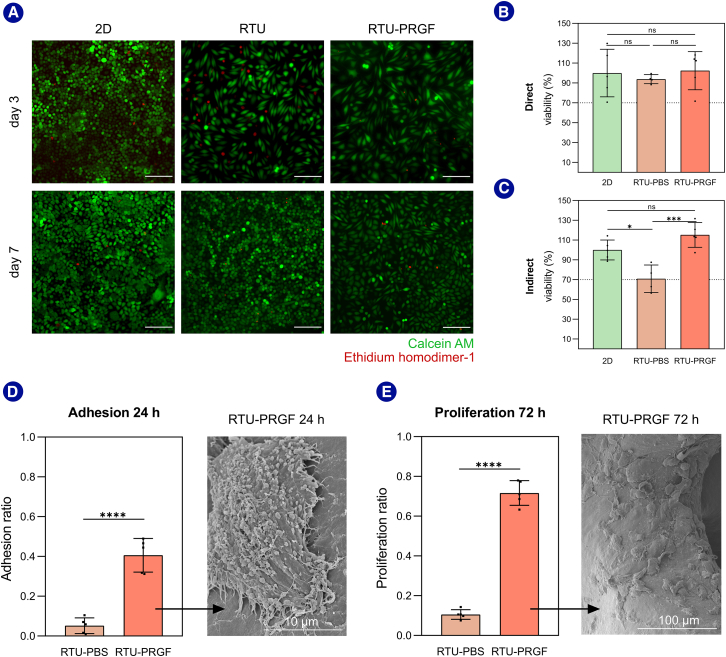
Cell-scaffold interaction (A) Live/Dead staining of L-929 fibroblast cultures after 3 and 7 days in 2D conditions and on top of RTU scaffolds rehydrated with PBS and PRGF, respectively (scale bars, 100 μm). Biocompatibility assessment following ISO 10993 standards, evaluated by both direct contact (B) and extract-based (C) assays on L-929 fibroblasts. Cell adhesion and proliferation: SEM imaging and quantification of HDF fibroblast adhesion 24 h post-seeding on the scaffolds (D), followed by proliferation assessment after 72 h (E). Statistical significance is indicated as follows: not significant (ns); ∗*p* < 0.05, ∗∗*p* < 0.01, ∗∗∗*p* < 0.001, and ∗∗∗∗*p* < 0.0001.

Live/Dead staining was carried out at days 3 and 7 after cell seeding (Figure 4A). Calcein-AM (green) and ethidium homodimer-1 (red) fluorescent labeling revealed high percentages of viable cells in all experimental conditions. Cell viability on both RTU-PBS and RTU-PRGF scaffolds appeared similar to the positive control, with few dead cells observed across all groups. By day 7, all scaffolds showed an apparent increase in cell density, suggesting active cell proliferation. These observations are qualitative only, based on Live/Dead staining images, while quantitative cell viability analysis is presented in the following paragraph. Overall, the fluorescent images confirmed that both RTU-PBS scaffolds and RTU-PRGF scaffolds maintained cell viability for at least 7 days, regardless of the rehydration medium, demonstrating their cytocompatibility and potential for skin tissue engineering applications.

To further confirm these findings, quantitative direct and indirect cytotoxicity assays were performed according to the ISO 10993 guideline for “Biological evaluation of medical devices,” which establishes a threshold of ≥70% viability relative to untreated controls for a material to be considered non-cytotoxic. In the direct assay (Figure 4B), L-929 fibroblasts were seeded directly onto the scaffolds, while in the indirect assay (Figure 4C), cells were cultured with scaffold extracts. In both cases, all formulations exhibited viability values above 80%. No statistically significant differences were observed between the groups in the direct assay. However, in the indirect assay, RTU-PRGF scaffolds showed significantly higher cell viability (115.27%) than RTU-PBS scaffolds (70.90%), suggesting a beneficial effect of the PRGFs on cytocompatibility.

Altogether, these results demonstrate that all tested RTU scaffolds meet the ISO-defined cytocompatibility criteria and can therefore be considered non-cytotoxic, justifying their use in further *in vitro* applications.

#### Cell adhesion and proliferation

To assess RTU scaffold-cell interactions, cell adhesion and proliferation assays were conducted at 24 and 72 h, respectively, using HDFs, primary cells isolated from human skin explants from adult donors and cryopreserved at passage one. HDF cell adhesion after 24 h was significantly improved in RTU-PRGF scaffolds compared to RTU-PBS scaffolds (Figure 4D, left), likely due to the presence of bioactive molecules such as fibronectin, PDGF, and IGF-1, which are known to promote cell attachment.^77^ Previous experiments^77^ had shown that cell attachment to the scaffolds was slow, and shorter incubation times yielded very weak absorbance signals in the WST-1 assay, likely due to insufficient adhesion at the time of measurement. Therefore, 24-h incubation was selected, acknowledging that some degree of proliferation may have occurred, but prioritizing the assurance of proper cell attachment. Both RTU-PBS and RTU-PRGF scaffolds showed improved cell adhesion compared to cells seeded directly onto plastic wells. These observations are consistent with previous studies reporting that gelatin, due to the presence of RGD sequences in its polymeric structure, promotes cell adhesion and proliferation.^71^ Notably, a significant difference (*p* < 0.0001) was observed in the RTU-PRGF scaffold group, which exhibited an enhanced capacity to promote cell adhesion. Cell proliferation, quantified at 72 h (Figure 4E, left), was also markedly higher in the RTU-PRGF group (*p* < 0.0001), indicating that PRGFs not only facilitates initial cell attachment but also provides a favorable biochemical milieu for sustained cell growth.

SEM was performed to further confirm the interactions between HDFs and the scaffold surface. After 24 h of incubation, SEM images revealed individual HDFs firmly attached and well spread on the surface of RTU-PRGF scaffolds, indicating early cell adhesion (Figure 4D, right). After 72 h, SEM showed a notable increase in the number of attached cells, with extensive cell spreading and coverage, as well as signs of ECM deposition (Figure 4E, right). These results confirm progressive cell colonization and active interaction with the biomaterial over time.^78^

Together, these results demonstrate that PRGFs enhance the biological performance of RTU gelatin-alginate-based scaffolds, indicating their potential suitability for therapeutic development. It has been shown that PRGFs could improve cell viability, promote adhesion and proliferation, and facilitate robust cell-material interactions, which are key features for tissue engineering applications.^79^

### *Ex vivo* studies

**Figure 5:**
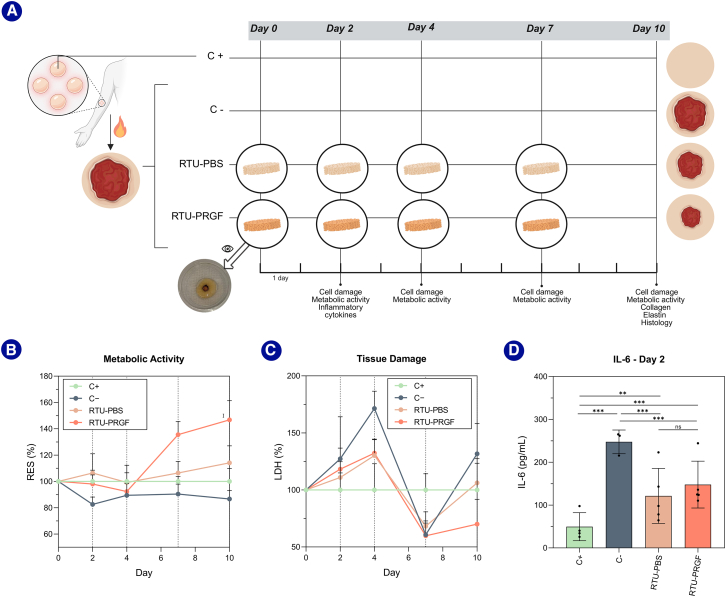
*Ex vivo* skin explant study using human organotypic skin explant cultures (hOSECs) (A) Experimental design of the cutaneous burn model assay, outlining the workflow and conditions for scaffold evaluation. (B–C) Cell viability assessment, measuring cell damage (LDH%) (B) and metabolic activity (RES%) (C) of skin cells across four experimental groups: healthy skin (C+), burn injury (C−), burn & RTU-PBS scaffold (RTU-PBS), and burn & RTU-PRGF scaffold (RTU-PRGF). (D) Inflammatory response, analyzing the effect of the burn condition on the synthesis of the pro-inflammatory cytokine IL-6 after 48 h in the same experimental groups (C+, C−, RTU-PBS, and RTU-PRGF). Statistical significance is indicated as follows: not significant (ns); ∗*p* < 0.05, ∗∗*p* < 0.01, ∗∗∗*p* < 0.001, and ∗∗∗∗*p* < 0.0001. See also Figure S1.

Human organotypic skin explant cultures (hOSECs) were employed as an *ex vivo* model to evaluate the therapeutic potential of RTU scaffolds during a 10-day wound healing period. Human organotypic skin explants offer a physiologically relevant *ex vivo* model that preserves the full architecture of human skin, including epidermis, dermis, appendages, and resident immune cells. Unlike traditional *in vitro* or animal models, they better replicate the complexity of human skin and wound healing dynamics.^80^^,^^81^ This makes them highly suitable for evaluating cytocompatibility, therapeutic efficacy, and delivery systems under near-clinical conditions. Additionally, they are ethically acceptable and cost-effective, yet remain underutilized in skin research.^82^^,^^83^ Among *ex vivo* models, hOSECs represent a highly advanced and physiologically relevant platform, preserving full-thickness architecture, viability, and epidermal-dermal crosstalk. Cultured at the air-liquid interface, they enable realistic testing of biomaterials, wound dressings, and therapeutic compounds under near-clinical conditions.^84^ The experimental design is summarized in Figure 5A, where samples were analyzed at multiple time points to monitor tissue damage, metabolic activity, inflammatory response, ECM remodeling, and histological regeneration.

As shown in Figure 5B, the resazurin reduction assay, which reflects cellular metabolic activity, indicated a progressive increase in metabolic activity in the RTU-PRGF group, with values surpassing both C− and C+ conditions from day 4 onward, highlighting the RTU-PRGF scaffold’s ability to promote cellular viability and functional activity in damaged tissue. In parallel, lactate dehydrogenase (LDH) release used as a marker of tissue damage^31^^,^^85^ peaked at day 2 in the negative control (C−), while the treated groups (RTU-PBS and RTU-PRGF) displayed reduced LDH levels throughout the study, suggesting lower cytotoxicity and enhanced tissue preservation (Figure 5C). The anti-inflammatory effect was evaluated by quantifying interleukin (IL)-6 in culture supernatants at day 2. This cytokine was markedly elevated in the C− group (247.56 pg/mL), confirming an acute inflammatory response. In contrast, both treated groups exhibited significantly lower IL-6 levels compared to C−, with 121.35 pg/mL for RTU-PBS and 147.85 pg/mL for RTU-PRGF, approaching those of the healthy control (C+), thereby indicating a strong immunomodulatory effect (Figure 5D).

**Figure 6:**
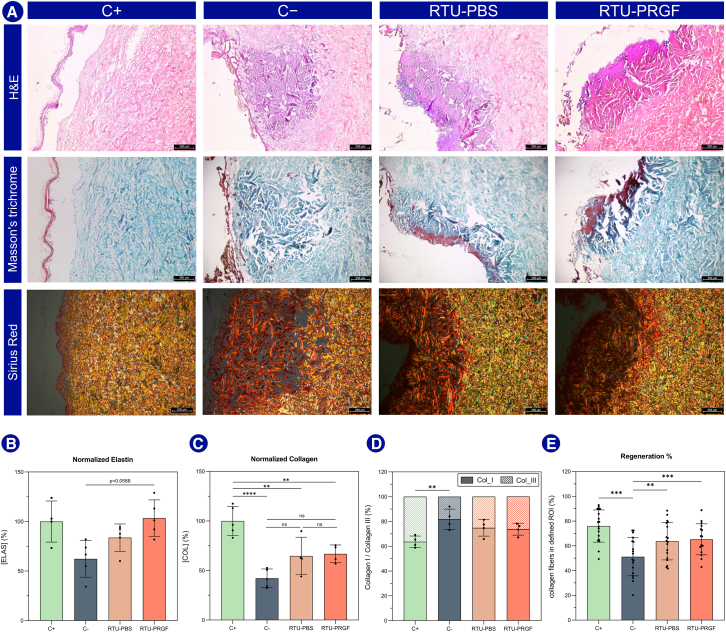
*Ex vivo* skin explant study using human organotypic skin explant cultures (hOSECs) (A) Histological analysis of skin explants stained with hematoxylin and eosin, Masson’s trichrome, and Sirius Red under polarized light, comparing the healthy skin (C+), burn injury 0 (C−), and burn & treatment (RTU-PBS and RTU-PRGF) groups (scale bars, 250 μm). (B) Elastin and (C) collagen quantification across the four experimental groups to assess extracellular matrix remodeling and tissue regeneration. (D) Collagen I/collagen III proportion in the papillary dermis. (E) Regeneration percentage based on the quantification of collagen fibers within the ROI of the wound. Scale bars, 250 μm. Statistical significance is indicated as follows: not significant (ns); ∗*p* < 0.05, ∗∗*p* < 0.01, ∗∗∗*p* < 0.001, and ∗∗∗∗*p* < 0.0001.

To assess matrix remodeling, histological staining was performed. Hematoxylin and eosin (H&E) staining was used to evaluate tissue architecture and healing progression, while Masson’s trichrome staining was used to examine the organization, distribution, and quality of dermal collagen fibers. In addition, Sirius Red combined with polarized light microscopy was used to specifically visualize and quantify collagen content. Although the most superficial layer, the epidermis, appeared detached in all conditions, likely due to sample handling or processing artifacts, this prevented any definitive assessment of epidermal regeneration. Notably, some epidermal remnants were visible in the RTU-PRGF-scaffold-treated group in the H&E staining (Figure 6A), but their continuity and structural integrity could not be reliably evaluated. Therefore, the analysis focused on the dermal compartment, which plays a central role in the structural restoration of wounded tissue. In this sense, both treated groups showed improved ECM remodeling within the dermis, with RTU-PRGF-scaffold-treated group exhibiting the most pronounced effect, as evidenced by the denser and more organized collagen network observed in both Masson’s trichrome and Sirius Red stainings (Figure 6A). These histological observations should be interpreted qualitatively, as imaging alone is not sufficient to demonstrate definitive superiority.

Our results demonstrate qualitatively that RTU-PRGF-treated skin exhibits a higher proportion of basophilic ECM areas compared to control and other treatment groups. This basophilia likely reflects an increased deposition of immature collagen (predominantly type III) and associated proteoglycans and glycosaminoglycans (GAGs), which are characteristic of early-stage tissue regeneration.^86^^,^^87^^,^^88^ These molecules, rich in acidic groups, retain basic dyes such as hematoxylin, producing the observed basophilic staining in histological sections. The predominance of immature collagen and ECM components in these areas is consistent with improved structural scaffold formation, which is critical for subsequent collagen maturation and skin remodeling. Overall, these findings support the notion that RTU-PRGF accelerates and enhances cutaneous regeneration, providing a favorable environment for tissue repair.

These qualitative observations were supported by quantitative analysis (Figures 6B and 6C), through elastin and collagen content quantification at day 10.^31^ Results revealed that both parameters were significantly increased in the treated groups (RTU-PBS and RTU-PRGF) compared to the wounded control group (C−). Elastin production was particularly higher in the RTU-PRGF scaffold group (103.4%), even surpassing levels observed in the healthy control (100%), suggesting more mature and functional tissue remodeling,^89^ while lower values were obtained for RTU-PBS (83.7%) and the negative control (62.3%).

In addition, the relative proportion of collagen I and III was assessed by image analysis of Sirius Red staining observed under polarized light in defined regions of interest (ROIs) within the papillary dermis (Figure 6D). Polarized light microscopy of Sirius-Red-stained sections revealed group-dependent differences in birefringent collagen fiber composition. Normal papillary dermis presented a relative proportion of collagen-type-I-associated fibers of 63.6% (standard deviation [SD] 4.6). In wounded dermis without treatment, this proportion increased substantially to 81.8% (SD 8.2). In contrast, treated wounds showed lower relative proportion of collagen I, with values of 74.8% (SD 6.6) in the RTU-PBS group and 73.8% (SD 4.8) in the RTU-PRGF group. Although all wounded samples demonstrated a shift toward a higher fraction of thick red-orange birefringent fibers compared with normal dermis, treatment attenuated the magnitude of this shift relative to untreated wounds.

Collagen type III is typically deposited early during wound healing as part of a provisional ECM and is progressively replaced by collagen type I during the remodeling phase. However, an excessive or premature increase in the collagen I/III proportion has been associated with dysregulated matrix remodeling and fibrotic scar formation rather than physiological regeneration.^90^ The markedly elevated collagen type I relative proportion observed in untreated wounds is therefore consistent with a pronounced shift toward a more mature collagen architecture.^91^ In contrast, treated wounds maintained a comparatively lower relative proportion of collagen type I, suggesting prolonged presence of collagen type III, as indicated by thin green-yellow birefringent fibers and a more gradual transition toward collagen type I. This collagen profile supports the interpretation that treatment modulates, rather than delays, dermal remodeling, promoting a matrix composition closer to that of normal dermis and potentially limiting fibrotic outcomes.

Finally, to further evaluate tissue regeneration, the area of newly formed collagen was quantified based on the percentage of relative intensity of stained collagen fibers within the studied ROI. Both RTU-PBS (63.68%) and RTU-PRGF (65.21%) groups showed a statistically significant increase in regeneration percentage compared to the untreated control (51.17%) (Figure 6E).

Taken together, these *ex vivo* results demonstrate that RTU scaffolds not only reduce tissue damage and inflammatory signaling but also support metabolic activity, ECM regeneration, and overall wound healing in human skin explants. Although full macroscopic wound closure was not observed due to epidermal loss in all groups, the data strongly suggest that RTU-PRGF scaffolds promote more robust and organized dermal regeneration, highlighting their therapeutic potential in enhancing deep tissue repair. Importantly, histological observations provide qualitative support but are complemented and reinforced by quantitative biomarker and molecular data, ensuring that interpretations of regenerative potential are grounded in robust, multi-level evidence rather than imaging alone.

To further contextualize the translational relevance of our findings, an additional exploratory *ex vivo* experiment was conducted using a commercially available wound care product (balsam formulation) under comparable experimental conditions and assessing the same quantitative parameters evaluated in the primary study. Although this supplementary analysis was performed independently from the original experimental series (which avoids statistical comparisons to be established) and did not include histological assessment, it serves as a practical reference point relative to a market-standard formulation. As shown in Figure S1, the commercial product displayed a biological response profile that qualitatively resembled the trends observed for RTU-PBS in terms of metabolic activity and tissue damage over time. In contrast, the RTU-PRGF group was characterized by consistently higher metabolic activity levels, suggesting a more pronounced regenerative stimulus under similar experimental settings. Regarding anti-inflammatory modulation, IL-6 measurements in the commercial product group showed a reduction trend; however, the magnitude of this effect appeared less marked than that observed in our treatment groups. With respect to ECM remodeling, both elastin and collagen levels in the commercial product group showed an improvement compared to its corresponding negative control (C−), following a trend consistent with that observed in our treatment groups in the primary experiment. The overall pattern of responses supports the notion that the RTU-PRGF system achieves a more favorable regenerative profile across multiple parameters when considered in parallel under comparable experimental frameworks.

Overall, in the present study, RTU-personalized gelatin-alginate-based scaffolds were successfully fabricated using 3D bioprinting technology and functionally enhanced through rehydration with patient’s PRGFs. Their ability to integrate with damaged skin tissue through the use of natural materials, personalized architectures, and autologous PRGFs underlines their clinical potential. As tissue engineering continues to progress, the development of accessible, customizable, and growth-factor-loaded scaffolds such as RTU-PRGF scaffolds will likely shape the future of advanced wound care, contributing to more effective therapies, reduced healthcare costs, and improved patient outcomes.

### Limitations of the study

While the present study provides promising results, several limitations should be considered within the context of ongoing translational development. First, the system has been evaluated at a preclinical stage, and its performance has not yet been assessed in long-term, immunocompetent, or *in vivo* wound models. Current experimental models only partially capture the complexity of human wounds, where interindividual variations in wound biochemistry, immune status, and ECM composition may influence scaffold performance. Although these aspects remain to be explored, they constitute well-defined next steps to further validate therapeutic efficacy under more physiologically relevant conditions.

Second, although the lyophilized RTU format provides clear advantages in terms of stability, storage, and handling, additional studies are required to confirm batch-to-batch reproducibility, sterility assurance, and robustness under Good Manufacturing Practice (GMP) conditions. These efforts, together with the simplification and standardization of manufacturing workflows, will be essential to ensure consistent product performance and facilitate clinical implementation.

Third, while the incorporation of autologous PRGFs represents a clinically attractive strategy, current approaches are based on a single administration. Future developments may benefit from more personalized or dynamically adaptable loading strategies that respond to the evolving physiological state of chronic wounds, enabling more precise modulation of the healing microenvironment. Regulatory and translational considerations also remain to be further clarified. The classification of combined acellular scaffolds with autologous biologics may vary across jurisdictions, and early alignment with regulatory frameworks will be important to streamline clinical translation.

Finally, the current system does not yet incorporate sensing or theranostic functionalities. The integration of diagnostic capabilities within RTU scaffolds represents a promising avenue for future research, with the potential to enable real-time monitoring of wound status and support more informed clinical decision-making. Overall, these limitations do not detract from the translational potential of the proposed system but rather define a clear and feasible roadmap for its continued development toward clinical application.

## Resource availability

### Lead contact

Requests for further information and resources should be directed to and will be fulfilled by the lead contact, Gorka Orive (gorka.orive@ehu.eus).

### Materials availability

This study did not generate new unique reagents.

### Data and code availability

• All data reported in this paper will be shared by the lead contact upon request.
• This article does not report original code.
• Any additional information required to reanalyze the data reported in this paper is available from the lead contact upon request.

## Acknowledgments

This work was funded by the Spanish 10.13039/501100010198Ministry of Economy, Industry, and Competitiveness (PID2022-139746OB-I00/AEI/10.13039/501100011033) and by the 10.13039/501100003451University of the Basque Country, through University-Business-Society Projects 2022 (US23/02). Lidia Maeso acknowledges the 10.13039/501100003086Basque Government for her Ph.D. grant (PRE_2022_1_0053). Sadegh Ghorbani acknowledges support from the 10.13039/501100009708Novo Nordisk Foundation (NNF22OC0073507). Schematic designs were created using BioRender.com.

## Author contributions

Conceptualization, L.M., M.H.A., and G.O.; investigation, L.M., R.T., E. Alonso, J.L., F.G., T.E.-d.-S., and M.N.; writing – original draft, L.M., R.T., E. Alonso, and T.E.-d.-S.; writing – review & editing: M.H.A., E. Alonso, J.L., J.Z., S.G., A.M., A.D.-P., and G.O.; funding acquisition, E. Anitua and G.O.

## Declaration of interests

The authors declare the following competing financial interests: E. Anitua serves as Scientific Director, and G.O., R.T., and M.H.A. are researchers at BTI Biotechnology Institute, a company specialized in dental implantology and PRGF-Endoret technology.

## STAR★Methods

### Key resources table

**Table:** 

REAGENT or RESOURCE	SOURCE	IDENTIFIER
**Biological samples**		

Human organotypic skin explant cultures (hOSECs)	GAIKER (https://www.gaiker.es/)	N/A

**Chemicals, peptides, and recombinant proteins**		

Gelatin from bovine skin (Type B, ∼225 g Bloom)	Sigma-Aldrich	SKU G9391
Calcium chloride (CaCl_2_)	Sigma-Aldrich	SKU 793639
Cell Proliferation Reagent WST-1 (4-[3,4-iodophenyl)-2-(4-nitrophenyl)-2H-5-tetrazolio]-1,3-benzenedisulfonate)	Sigma-Aldrich	SKU 5015944001
Resazurin reagent	Sigma-Aldrich	SKU R7017
Methylene blue	Sigma-Aldrich	SKU 159270
Alizarin red	Sigma-Aldrich	SKU A5533
Colagenasa from *Clostridium histolyticum* (C0130)	Sigma-Aldrich	SKU C9697
Dulbecco’s Modified Eagle’s Medium (DMEM)	Sigma-Aldrich	SKU D6429
Pharmaceutical-grade alginate	FMC Biopolymer (https://www.fmcbiopolymer.com/)	N/A
Picrosirius Red staining reagents	Epredia	https://www.epredia.com/es
Gill’s hematoxylin No. 2, 1% alcoholic eosin	Epredia	72504
Eagle’s Minimum Essential Medium (EMEM 30–2003)	ATCC	30–2003
Phosphate-buffered saline (PBS, pH 7.4, 1×)	Fisher Scientific	Cat# 10316743
Trypsin	Fisher Scientific	Cat# 11580626
Fetal bovine serum (FBS)	Fisher Scientific	Cat# 11543407
Penicillin–streptomycin solution	Fisher Scientific	Cat# 11548876
Fibroblast Medium (FM)	Innoprot	P60108
Human IL-6 DuoSet ELISA	Bio-Techne	DY206-05 R&D
Human IL-8/CXCL8 DuoSet ELISA	Bio-Techne	DY208-05 R&D

**Critical commercial assays**		

Cell Counting Kit-8 (CCK-8)	Sigma-Aldrich	SKU 96992
Masson’s trichrome staining kit	Labolan	010802
Pierce™ BCA Protein Assay Kit	Fisher Scientific	Cat# 23227
LIVE/DEAD® viability/cytotoxicity kit	Fisher Scientific	Cat# L3224
Lactate dehydrogenase (LDH) cytotoxicity assay	Promega	Cat# J2380
Fastin™ Elastin kit	Biocolor	F2000
Sircol™ Collagen kit	Biocolor	S2000

**Experimental models: Cell lines**		

Mouse L-929 fibroblasts	ATCC	CCL-1 ™
Human dermal fibroblasts (HDFs)	Innoprot	P10858

**Software and algorithms**		

ImageJ	Schneider et al.^92^	https://imagej.nih.gov/ij/
Biorender	Biorender	https://Biorender.com
R: A Language and Environment for Statistical Computing.	R Core Team^93^	https://www.r-project.org/
Adobe illustrator	Adobe	https://www.adobe.com/products/illustrator.html

### Experimental model and study participant details

#### Human organotypic skin explant cultures (hOSECs)

Full-thickness human skin samples were ethically obtained from cosmetic surgery procedures such as abdominoplasties, with prior informed consent and authorization granted by French Government Ethical Committee according to the French law L.1245 CSP. Abdominal human organotypic skin explant samples were obtained from a patient (44-years-old) with a body mass index of 33 that presented phototype II and no scar marks. The skin samples were cut (0.8 cm2 pieces) and placed in culture plates in a culture medium with the epidermis facing upward (MIL218C, Biopredic International, Saint Gregoire, France). Upon collection, tissues were transported under sterile cold conditions and processed immediately. The skin samples were cleaned, trimmed, and standardized thermal wounds were created to simulate open burn injuries. The burn model was generated by exposure (20 s) to a soldering iron welder (1.8 mm diameter) that was previously heated (200°C), thus creating a 2nd-degree burn simulation (having epidermal and dermal layers of the skin affected). Wounded explants were placed on cell culture inserts in multi-well plates to maintain an air–liquid interface, preserving the structural and functional integrity of both the epidermis and dermis. Dulbecco’s modified Eagle’s medium (DMEM) (Sigma-Aldrich, Madrid, Spain) supplemented with antibiotics was added to the basal compartment and refreshed regularly.

#### PRGF

For PRGF obtention, peripheral venous blood was collected under aseptic conditions from three patients into 9 mL sodium citrate-containing tubes (3.8% w/v; BTI, Vitoria, Spain), after obtaining written informed consent and approval from the ethics committee (BTIIMD-02-IV-23-NF).

#### L-929 and human dermal fibroblasts (HDF)

Mice-derived L-929 fibroblasts (ATCC, CCL-(1) and Human dermal fibroblasts (HDFs; P10858, Innoprot) were used for *in vitro* cell studies. Cells were cultured in EMEM 30–2003 medium (ATCC) complemented with 10% FBS (v/v) and 1% penicillin-streptomycin (v/v) (Fischer Scientific) and complete Fibroblast Medium (FM; P60108, Innoprot), according to the supplier’s recommendations., respectively. Cell cultures were incubated at 37°C with 5% CO2 and humidified-controlled environment and cell division was regularly performed when cells achieved confluence.

#### Ethics statement

Peripheral venous blood for PRGF preparation was collected under aseptic conditions following written informed consent and approval from the ethics committee (BTIIMD-02-IV-23-NF). Human skin samples were obtained from a commercial provider compliant with the French Public Health Code (Article L1245), collected with informed consent, fully anonymized, and not requiring additional ethical approval according to national regulations.

### Method details

#### Preparation of alginate–gelatin bioinks

Four distinct alginate–gelatin-based bioink formulations were prepared, with the specific concentrations for each formulation detailed in Table 1. For each formulation, alginate and gelatin powders were weighed and dissolved in phosphate-buffered saline (PBS, 20 mL) under continuous magnetic stirring (60°C, 1 h). After complete dissolution, the resulting bioinks were stored overnight (4°C) to ensure full hydration and used for 3D bioprinting the following day (Figure S2).

**Table 1:** Composition details of selected alginate-gelatin bioink formulations

F1	F2	F3	F4
5% (w_solid_/v_PBS_)		8% (w_solid_/v_PBS_)	
1:1	2:1	1:1	2:1
0.5 g gel (2.5%)	0.67 g gel (3.33%)	0.8 g gel (4%)	1.07 g gel (5.33%)
0.5 g alg (2.5%)	0.33 g alg (1.66%)	0.8 g alg (4%)	0.53 g alg (2.66%)
20 mL PBS	20 mL PBS	20 mL PBS	20 mL PBS

##### 3D bioprinting and post-processing of RTU scaffolds

RTU alginate–gelatin-based scaffolds were fabricated using a CELLINK BIO X bioprinter (CELLINK, Sweden). The selected four bioink formulations were tested to identify the most suitable composition for 3D printing. Preliminary printing trials were performed to optimize key parameters, and the optimal conditions were established as follows: 27G conical needle, printing speed of 9 mm s^−1^, and 30% infill. Additionally, the inks were preheated at 37°C for 20 min prior to printing. Printability was calculated following Equation 1:

(Equation 1) Printability(%)=realarea(Ai)/idealarea(A)∗100,

The bioprinted hydrogels were crosslinked with CaCl_2_ solution (100 mM, prepared by dissolving 2.22 g CaCl_2_ in 200 mL Milli-Q water). Finally, scaffolds obtained from formulation F3 were lyophilized (42 h) and stored until use (−20°C).

#### PRGF isolation and preparation of RTU-Personalized scaffolds

PRGF was prepared at room temperature (RT) following the manufacturer’s instructions (Endoret System, BTI Biotechnology Institute, S.L., Vitoria, Spain). Blood samples were immediately centrifuged (BTI System IV; 580 × g, 8 min), resulting in the separation of three layers: (i) a lower red blood cell layer, (ii) an intermediate leukocyte-rich buffy coat, and (iii) an upper plasma layer with a gradient of platelet concentration. The upper plasma fraction just above the buffy coat (∼2 mL per tube) was carefully collected to obtain a leukocyte-free PRGF fraction, minimizing pro-inflammatory responses. Liquid PRGF was transferred to sterile tubes and activated by adding CaCl_2_ (10%, 50 μL/mL plasma), to induce platelet degranulation and the release of growth factors such as PDGF, TGF-β, VEGF, IGF-1, HGF, FGFb, or EGF. Following clot formation and retraction, the PRGF supernatant was filtered using a sterile syringe filter with a 0.22 μm pore size hydrophilic PES membrane (polyethersulfone) and stored until use (−80°C). RTU-personalized scaffolds were obtained by rehydrating RTU lyophilized scaffolds with PRGF solution (200 μL, 90 min, 37°C) to enable growth factor adsorption.

#### Rheological characterization

The rheological properties of the F3 bioink and the hydrogel were evaluated (Discovery Hybrid Rheometer HR-2, TA Instruments, 8 mm parallel plate geometry, gap distance of 1000 μm, 25°C). The base BioInk, referred to as the non-crosslinked BioInk (Non-CL-Ink), was used as the test sample. Rheological behavior was characterized by performing frequency sweep tests (0.1–400 rad/s, at 1% strain) to assess complex viscosity, storage modulus (G′), and loss modulus (G″). Amplitude sweeps were conducted at a fixed angular frequency (10 rad s^−1^, 0.015%–555%). Flow behavior was also evaluated through shear rate sweeps to assess shear-thinning properties. Additionally, self-healing capacity was examined by applying time sweep tests (10 rads^−(1^) with alternating strain cycles (1% strain followed by three cycles of 100% strain, each separated by 300 s resting intervals). Recovery of rheological properties after each cycle was quantified to assess the hydrogel’s self-healing performance.

#### Mechanical compressive properties

Compression tests of hydrogels were performed using a universal testing machine (Instron 5967, UK) equipped with a 500 N load cell at a crosshead speed of 0.5 mm min^−1^. Dimensions of each specimen were measured with a digital caliper prior to testing. Hydrogels were prepared by preheating the ink (37°C, 20 min), casting into cylindrical molds (∼100 μL, ∼4.7 mm diameter, ∼4.7–5 mm height), and refrigerating at 4°C for at least 3 h. Two conditions were evaluated: (i) non-crosslinked gel–alginate bioink (Non-CL-Ink), and (ii) *in situ* crosslinked bioink (CL-inSitu) with 100 mM CaCl_2_ solution. Prior to testing, samples were placed on ice and individually demolded into the corresponding solution (15 min, RT). The compressive Young’s modulus was calculated from the linear region of the stress–strain curve (15–25% strain). Compressive stress at 40% strain, ultimate compressive strength (UTS), breaking point, and toughness (area under the curve) were also recorded.

#### Fourier transform infrared (FTIR) spectroscopy

Fourier transform infrared (FTIR) spectroscopy (PerkinElmer Spectrum 100) was performed to investigate the formation of the F3 gelatin-alginate-based scaffold. First, background subtraction was performed. Subsequently, transmittance spectra were collected (80% gauge, spectral range 4000–550 cm^−1^, resolution 4 cm^−1^, 10 scans). Ultimately, PerkinElmer Spectrum software was employed to proceed with the baseline correction and normalization.

#### Thermal gravimetric analysis (TGA)

Thermal properties of the gelatin–alginate-based scaffold were analyzed using a thermogravimetric analyzer (TGA, Q500, USA) from 30°C to 880°C at a heating rate of 10°C min^−1^ under a nitrogen atmosphere. All samples were freeze-dried prior to analysis.

#### Scanning electron microscopy (SEM)

The morphology and pore size of F3-based scaffolds were analyzed by SEM equipped with a field emission gun (FEI Quanta 200 ESEM FEG, USA; 5–10 kV, 5 mA). To preserve structural integrity and prevent pore collapse, samples were embedded in Milli-Q water prior to freezing (−20°C for 4 h, then transferred to −80°C overnight). Subsequently, samples were lyophilized (42 h), fractured in liquid nitrogen to expose the cross-section, and sputter-coated with a 10 nm gold layer (Quorum Q150T coater, UK). Pore size distribution was quantified using ImageJ software (National Institutes of Health, NIH).

#### Swelling behavior and degradation performance

Swelling behavior of the RTU 3D-bioprinted scaffolds was evaluated by immersing the dried F3-based samples in phosphate-buffered saline (PBS) under continuous agitation (300 rpm, 37°C). Prior to immersion, the initial dry weight of each sample was recorded. At predefined time points (10, 30, 60 and 90 min; 3, 6, and 24 h; 3 and 7 days), samples were removed, gently blotted to eliminate surface liquid, and weighed to determine their swollen mass. The swelling ratio was calculated using the following Equation 2:

(Equation 2) $$Swelling\;Ratio=\left(W_{s}{-W}_{o}\right)/W_{o}\text{,}$$

where *W*_*s*_ represents the swollen weight and *W*_*o*_ the original dry weight of the same sample.

Hydrolytic degradation of the scaffolds was evaluated under physiological-like conditions using lyophilized samples prepared from F3. Dried samples were weighed to determine their initial mass and then placed into labeled well plates containing an excess volume of PBS (pH 7.4, ∼1 mL per sample). The containers were sealed with lids and parafilm to prevent PBS evaporation and incubated (37°C). At predetermined time points (6 h, 1 day, 3 days, 1 week, 2 weeks and 3 weeks), individual samples were removed, lyophilized again, and their dry weight was recorded. The extent of degradation was assessed by comparing the weight of the remaining dry material at each time point with the initial freeze-dried weight.

In addition, enzymatic degradation was assessed to better simulate physiological matrix remodeling conditions. Briefly, samples were incubated at 37°C in a 0.1 M Tris buffer solution (pH 7.(4) containing 0.05 M CaCl_2_ and 20 U/mL collagenase (Sigma-Aldrich, #C0130). Degradation was evaluated at 1 h, 3 h, 6 h, 1 day, 3 days, 1 week, 2 weeks, and 3 weeks, matching the time points analyzed for hydrolytic degradation in PBS. At each time point, samples were collected, lyophilized, and weighed to determine mass loss as described above.

#### PRGF release study

To evaluate protein release from the RTU and RTU-PRGF scaffolds (*n* = 3), they were respectively rehydrated (37°C, 90 min) with PBS (200 μL, control group) and PRGF supernatant (200 μL, experimental group). PRGF loading efficiency was determined by gravimetric analysis following the rehydration process. Loading efficiency was calculated as the ratio between the retained PRGF volume and the initially applied volume (200 μL), expressed as a percentage. Following rehydration, the scaffolds were transferred to new wells containing fresh PBS (200 μL, 37°C). At predetermined time points (0, 2, 4, 7, and 10 days), the medium from each well was collected and stored for subsequent protein release quantification analysis (Pierce BCA Protein Assay Kit, Thermo Scientific, Cat. No. 23225), following the manufacturer’s instructions.

#### Cell viability study

Live/Dead staining was performed on L-929 cell-seeded RTU and RTU-PRGF scaffolds at days 3 and 7 using a Live/Dead viability kit (Life Technologies), following the manufacturer’s instructions. Calcein-AM and ethidium homodimer-1 were used to stain live (green fluorescence) and dead (red fluorescence) cells, respectively. Six samples per condition (scaffold + PBS and scaffold + PRGF) were analyzed, and cells cultured in standard 2D well plates were used as controls. Fluorescence images were captured (Nikon TMS inverted fluorescence microscope) to qualitatively assess cell viability and distribution within the scaffolds.

#### Biocompatibility study

The biocompatibility of the RTU and RTU-PRGF scaffolds was evaluated using L-929 mouse fibroblasts, following ISO 10993 guidelines and the protocol described by Echave et al.^76^^,^^94^ Cells were cultured in EMEM 30–2003 supplemented with 10% FBS (v/v) and 1% penicillin-streptomycin (v/v) at 37°C and 5% CO_2_. Both direct (cells seeded onto the scaffolds) and indirect (cells exposed to scaffold extracts) cytotoxicity tests were performed. Six samples per condition (scaffold + PBS and scaffold + PRGF) were analyzed, and cells cultured in standard 2D well plates were used as controls. Based on established protocols in tissue engineering,^94^^,^^95^ cells were incubated for 24 h prior to exposure to the scaffold extracts or direct contact tests to allow proper cell adhesion before cytotoxicity evaluation. Following this initial culture period, cells were exposed to the scaffolds and cultured for an additional 24 h, after which cell viability was assessed. Cellular metabolic activity, as an indicator of viability, was assessed using the CCK-8 assay, measuring absorbance at 450 nm with with a Tecan Infinite M2000 microplate reader. Results were normalized to untreated control cells, considered as 100% viable.

#### Cell adhesion and proliferation studie*s*

RTU and RTU-PRGF scaffolds (previously rehydrated in PBS and in PRGF, respectively) were placed into 48-well plates and seeded (30,000 cells/cm^2^ in 200 μL of culture medium) with HDFs (passage 2; 3,2 doublings). Cells seeded directly onto plastic wells served as positive controls.

For the adhesion assay, samples were incubated (24 h, 37°C, 5% CO_2_) and transferred to new wells containing fresh medium (200 μL); thereafter WST-1 reagent was added (Sigma-Aldrich, Cat. No. 5015944001; 1:10 dilution). After incubation (2 h), supernatant was transferred (150 μL) to a 96-well plate, and absorbance was measured (450 nm and 620 nm). Viability was calculated as Equation 3:

(Equation 3) Δ=[450(sample−blank)]−[620(sample−blank)],

For the proliferation assay, a similar setup was followed. After initial culture (24 h), the scaffolds were transferred to new wells with fresh medium to avoid interference from plastic-adhered cells. The samples were then maintained in culture for an additional 48 h (total of 72 h), under the same conditions. At the endpoint, the WST-1 assay was conducted following the same protocol described above. Due to the higher cell density, the samples were incubated for 1 h, after which absorbance was measured as previously described.

Both adhesion and proliferation samples, as well as control scaffolds without cells, were fixed and further analyzed by SEM to evaluate cell morphology and surface colonization (Figure S3).

#### *Ex vivo* studies with human organotypic skin explant cultures (hOSECs)

To evaluate the safety and therapeutic efficacy of the RTU-PRGF scaffolds under investigation, human organotypic skin explant cultures (hOSECs) were used as an *ex vivo* experimental model.

Skin explant samples were randomly assigned to four experimental groups (*n* = 5 each): (i) positive control (C+; healthy, unwounded, untreated skin), (ii) negative control (C−; wounded, untreated skin), (iii) RTU-PBS scaffold group (RTU-PBS; wounded skin treated with RTU-PBS scaffolds), and (iv) RTU-PRGF scaffold group (RTU-PRGF; wounded skin treated with RTU-PRGF scaffolds). To simulate a realistic clinical application, RTU scaffolds were rehydrated with either PBS or liquid PRGF (200 μL) and incubated for 1.5 h at 37°C and 5% CO_2_ prior to application. Both RTU-PBS and RTU-PRGF scaffolds were then carefully placed onto the wound site of each explant using sterile tweezers. Scaffolds were replaced every 48 h.

Explants were maintained under standard culture conditions (37°C, 5% CO_2_) for a total of 10 days. Throughout this time, the progression of tissue response was monitored by measuring lactate dehydrogenase (LDH) release (LDH Promega, Madrid, Spain) and resazurin reduction (Sigma-Aldrich) as indicators of cytotoxicity and metabolic activity, respectively. Anti-inflammatory efficacy was assessed by quantifying pro-inflammatory cytokine IL-6 in culture supernatants (ELISA, 48 h post-treatment). Additionally, to evaluate therapeutic efficacy, collagen and elastin content in the skin tissue was measured at the experimental endpoint after tissue digestion as indicators of extracellular matrix remodeling. This was done using commercially available Sircol Collagen and Fastin Elastin kits respectively, following the manufactureŕs instructions (Biocolor, Carrickfergus, UK). Results were expressed as collagen or elastin μg/tissue mg.

Histological analysis was performed on paraffin-embedded tissue sections fixed in 4% formaldehyde and cut at a thickness of 4 μm. Sections were deparaffinized in Citrosol and rehydrated through a graded ethanol series to distilled water. The following stainings were then carried out according to standardized protocols: (i) Hematoxylin–eosin (H&E): staining with Gill’s hematoxylin No. 2 (Epredia, the Netherlands) for 4 min, followed by counterstaining with 1% alcoholic eosin (Epredia, the Netherlands) for 30 s; (ii) Masson’s trichrome: staining with Groat’s hematoxylin for 4 min, rinsing in running tap water for 5 min, sequential staining with Fuchsin–Ponceau for 5 min, quick rinsing in acetic water by immersing and removing the slides once or twice, and subsequent staining with Molybdic Orange G for 5 min. After another quick rinse in acetic water, staining with Light Green was performed for 5 min, followed by a final quick rinse in acetic water; (iii) Picrosirius Red: incubation in fast green (0.03%) in picric acid for 30 min, rinsing with clear water, incubation with Sirius red (0.1%) in picric acid for 30 min, and washing with distilled water. After each staining, sections were dehydrated, cleared in Citrosol, and mounted with DPX. Sections stained with Picrosirius Red were also examined under polarized light to evaluate collagen organization and birefringence. For each sample, a representative image was acquired using a light microscope (LEICA DM2700 M). All images were coded and analyzed in a blinded manner using Fiji (ImageJ, version X; NIH, USA). Regions of interest (ROIs) were defined in the papillary dermis. For collagen quantification, color thresholding was performed on Sirius Red–stained dermal sections imaged under polarized light using predefined HUE ranges to discriminate birefringent collagen fiber populations. HUE thresholds were empirically determined using normal papillary dermis as an internal reference to encompass the full spectrum of birefringence corresponding to thick red–orange and thin green–yellow collagen fibers under our optical conditions. Collagen type I, identified by red–orange birefringent fibers, was segmented using a HUE range of 0–20, whereas collagen type III, characterized by green–yellow birefringence, was segmented using a HUE range of 25–160. Identical threshold settings were applied to all images to ensure consistency across samples. For each image, three ROIs were analyzed, and collagen content was quantified as the percentage of the area exceeding the threshold (% area positive). The relative proportion of type I and type III collagen in each image was calculated as Equations 4 and 5:

(Equation 4) ProportionofCollagenI=AreaColI/AreaColI+AreaColIII,

(Equation 5) $$Proportion\;of\;Collagen\;III=Area\;Col\frac{III}{Area}Col\;I+Area\;Col\;III\text{,}$$

### Quantification and statistical analysis

Data were analyzed using appropriate parametric or non-parametric tests based on the distribution and homogeneity of variances. Distributional assumptions required for parametric statistical tests were assessed using a combination of methods, including visual inspection, the Shapiro–Wilk test for normality, and Levene’s test for homoscedasticity. Comparisons between a single group and a reference value, or between two independent groups, were performed using the Student’s *t* test. When the assumption of equal variances was not fullfilled, Welch’s *t* test was computed. In situations where normality assumptions were not satisfied, the Wilcoxon rank-sum test was applied to compare two independent groups. For comparisons involving more than two groups, one-way analysis of variance (ANOVA) was performed, followed by Tukey’s Honestly Significant Difference (HSD) test for post hoc multiple comparisons. When the assumption of homogeneity of variances was violated, Welch’s ANOVA was used as an alternative to the standard one-way ANOVA, followed by Games–Howell post hoc testing for pairwise group comparisons. The Kruskal–Wallis test was used for comparing multiple groups under non-parametric conditions with Dunn’s test applied to identify significant differences between groups. Statistical analyses were performed in R.^93^ Statistical significance is indicated as follows: not significant (ns), *p* < 0.05 (∗), *p* < 0.01 (∗∗), *p* < 0.001 (∗∗∗), and *p* < 0.0001 (∗∗∗∗).

## References

[1] Sen C.K., Gordillo G.M., Roy S., Kirsner R., Lambert L., Hunt T.K., Gottrup F., Gurtner G.C., Longaker M.T. (2009). Human skin wounds: A major and snowballing threat to public health and the economy. Wound Repair Regen..

[2] Abuhamad A.Y., Masri S., Fadilah N.I.M., Alamassi M.N., Maarof M., Fauzi M.B. (2024). Application of 3D-Printed Bioinks in Chronic Wound Healing: A Scoping Review. Polymers.

[3] Sen C.K. (2019). Human Wounds and Its Burden: An Updated Compendium of Estimates. Adv. Wound Care.

[4] Sen C.K. (2025). Human Wound and Its Burden: Updated 2025 Compendium of Estimates. Adv. Wound Care.

[5] Queen D., Harding K. (2024). Estimating the cost of wounds both nationally and regionally within the top 10 highest spenders. Int. Wound J..

[6] Maeso L., Antezana P.E., Hvozda Arana A.G., Evelson P.A., Orive G., Desimone M.F. (2024). Progress in the Use of Hydrogels for Antioxidant Delivery in Skin Wounds. Pharmaceutics.

[7] Liang Y., He J., Guo B. (2021). Functional Hydrogels as Wound Dressing to Enhance Wound Healing. ACS Nano.

[8] Azizi R., Kermanian M., Alinezhad V., Kalantari-Hesari A., Yousefiasl S., Maeso L., Orive G., Mohammadi A., Esmaeilzadeh K., Seyedhamzeh M. (2025). An intelligent ZIF-based nanoplatform for photothermal/chemodynamic-induced combination therapy with O2 evolution properties for improved infected wound regeneration. J. Mater. Chem. B.

[9] Theocharidis G., Yuk H., Roh H., Wang L., Mezghani I., Wu J., Kafanas A., Contreras M., Sumpio B., Li Z. (2022). A strain-programmed patch for the healing of diabetic wounds. Nat. Biomed. Eng..

[10] Jiang Y., Trotsyuk A.A., Niu S., Henn D., Chen K., Shih C.C., Larson M.R., Mermin-Bunnell A.M., Mittal S., Lai J.C. (2023). Wireless, closed-loop, smart bandage with integrated sensors and stimulators for advanced wound care and accelerated healing. Nat. Biotechnol..

[11] Lavik E., Langer R. (2004). Tissue engineering: Current state and perspectives. Appl. Microbiol. Biotechnol..

[12] Alinezhad V., Ghodsi R., Bagheri H., Beram F.M., Zeighami H., Kalantari-Hesari A., Salarilak L., Mostafavi E., Ahmadian Z., Shahbazi M.A., Maleki A. (2024). Antioxidant, hemostatic, and injectable hydrogels with photothermal antibacterial activity to accelerate full-thickness wound regeneration. New J. Chem..

[13] Lukin I., Erezuma I., Maeso L., Zarate J., Desimone M.F., Al-Tel T.H., Dolatshahi-Pirouz A., Orive G. (2022). Progress in Gelatin as Biomaterial for Tissue Engineering. Pharmaceutics.

[14] Zhou X., Yu X., You T., Zhao B., Dong L., Huang C., Zhou X., Xing M., Qian W., Luo G. (2024). 3D Printing-Based Hydrogel Dressings for Wound Healing. Adv. Sci..

[15] Shi S., Hu L., Hu D., Ou X., Huang Y. (2024). Emerging Nanotherapeutic Approaches for Diabetic Wound Healing. Int. J. Nanomed..

[16] Meng X., Xiao X., Jeon S., Cho D.S., Zhang K., Kwon Y.H., Mo H., Park Y., Park B.-J., Kim D. (2025). Self-contracting, battery-free triboelectric wound healing strip with strong wet adhesion. Nat. Commun..

[17] Zhang Y.S., Dolatshahi-Pirouz A., Orive G. (2024). Regenerative cell therapy with 3D bioprinting. Science.

[18] Naghieh S., Chen X. (2021). Printability–A key issue in extrusion-based bioprinting. J. Pharm. Anal..

[19] Lee K.Y., Mooney D.J. (2012). Alginate: Properties and biomedical applications. Prog. Polym. Sci..

[20] FDA/Center for Drug Evaluation and Research (2025) Inactive Ingredient Search for Approved Drug Products.

[21] Malafaya P.B., Silva G.A., Reis R.L. (2007). Natural-origin polymers as carriers and scaffolds for biomolecules and cell delivery in tissue engineering applications. Adv. Drug Deliv. Rev..

[22] Aramwit P., Ågren M.S. (2016). Wound Healing Biomaterials.

[23] U.S. Food and Drug Administration (2021) 510(k) Premarket Notification: LUOFUCON PHMB Alginate Dressing.

[24] U.S. Food and Drug Administration (2009) 510(k) Premarket Notification: COLLASORB COLLAGEN WOUND DRESSING.

[25] U.S. Food and Drug Administration (1999) 510(k) Premarket Notification: DERMAPHYLYX HYDROPHILIC FOAM WOUND DRESSING.

[26] Wang Z., Yang J., Peng J., Zhu J., Li X., Du J., Cheng Y.Y., Xu J., Song F., Jia Z., Song K. (2025). A 3D printed biomimetic composite scaffold based on graphene/gelatin/sodium alginate bioink: Cell proliferation effects and toxicity assessments. J. Biomater. Appl..

[27] Chen J., Fan Y., Dong G., Zhou H., Du R., Tang X., Ying Y., Li J. (2023). Designing biomimetic scaffolds for skin tissue engineering. Biomater. Sci..

[28] Anitua E., Andí I., Sanchez M., Azofra J., del Mar Zalduendo M., de la Fuente M., Nurden P., Nurden A.T. (2005). Autologous preparations rich in growth factors promote proliferation and induce VEGF and HGF production by human tendon cells in culture. J. Orthop. Res..

[29] Anitua E., Sánchez M., Zalduendo M.M., De La Fuente M., Prado R., Orive G., Andía I. (2009). Fibroblastic response to treatment with different preparations rich in growth factors. Cell Prolif..

[30] Anitua E., Troya M., Goñi F., Gómez P., Tierno R., Pino A. (2020). A Novel Autologous Topical Serum Based on Plasma Rich in Growth Factors Technology Counteracts Ultraviolet Light-Derived Photo-Oxidative Stress. Skin Pharmacol. Physiol..

[31] Anitua E., Martinez Z., Arrue I., Gonzalez R., Tierno R., García A., Goñi F., Pino A. (2022). The Effect of an Autologous Protein-Based Topical Serum on Cutaneous Burns. J. Drugs Dermatol. JDD.

[32] Anitua E., Muñoz V., Aspe L., Tierno R., García-Salvador A., Goñi-de-Cerio F., Pino A. (2022). In vitro and in vivo Effect of Platelet-Rich Plasma-Based Autologous Topical Serum on Cutaneous Wound Healing. Skin Pharmacol. Physiol..

[33] Anitua E., Pino A., Jaen P., Orive G. (2016). Plasma Rich in Growth Factors Enhances Wound Healing and Protects from Photo-oxidative Stress in Dermal Fibroblasts and 3D Skin Models. Curr. Pharm. Biotechnol..

[34] Sievers J., Zimmermann R., Friedrichs J., Pette D., Limasale Y.D.P., Werner C., Welzel P.B. (2021). Customizing biohybrid cryogels to serve as ready-to-use delivery systems of signaling proteins. Biomaterials.

[35] Katsen-Globa A., Meiser I., Petrenko Y.A., Ivanov R.V., Lozinsky V.I., Zimmermann H., Petrenko A.Y. (2014). Towards ready-to-use 3-D scaffolds for regenerative medicine: Adhesion-based cryopreservation of human mesenchymal stem cells attached and spread within alginate-gelatin cryogel scaffolds. J. Mater. Sci. Mater. Med..

[36] Shin J., Choi S., Kim J.H., Cho J.H., Jin Y., Kim S., Min S., Kim S.K., Choi D., Cho S.W. (2019). Tissue Tapes—Phenolic Hyaluronic Acid Hydrogel Patches for Off-the-Shelf Therapy. Adv. Funct. Mater..

[37] Endres T., Zheng M., Beck-Broichsitter M., Kissel T. (2012). Lyophilised ready-to-use formulations of PEG-PCL-PEI nano-carriers for siRNA delivery. Int. J. Pharm. X..

[38] Özsoylu D., Isık T., Demir M.M., Schöning M.J., Wagner T. (2021). Cryopreservation of a cell-based biosensor chip modified with elastic polymer fibers enabling ready-to-use on-site applications. Biosens. Bioelectron..

[39] Baudequin T., Wee H., Cui Z., Ye H. (2023). Towards Ready-to-Use Iron-Crosslinked Alginate Beads as Mesenchymal Stem Cell Carriers. Bioengineering.

[40] Chen X.B., Fazel Anvari-Yazdi A., Duan X., Zimmerling A., Gharraei R., Sharma N.K., Sweilem S., Ning L. (2023). Biomaterials/bioinks and extrusion bioprinting. Bioact. Mater..

[41] Amorim P.A., d’Ávila M.A., Anand R., Moldenaers P., Van Puyvelde P., Bloemen V. (2021). Insights on shear rheology of inks for extrusion-based 3D bioprinting. Bioprinting.

[42] Schwab A., Levato R., D’Este M., Piluso S., Eglin D., Malda J. (2020). Printability and Shape Fidelity of Bioinks in 3D Bioprinting. Chem. Rev..

[43] Field E.H., Ratcliffe J., Johnson C.J., Binger K.J., Reynolds N.P. (2025). Self-healing, 3D printed bioinks from self-assembled peptide and alginate hybrid hydrogels. Biomater. Adv..

[44] Hao L., Zhao S., Hao S., He Y., Feng M., Zhou K., He Y., Yang J., Mao H., Gu Z. (2023). Functionalized gelatin-alginate based bioink with enhanced manufacturability and biomimicry for accelerating wound healing. Int. J. Biol. Macromol..

[45] Wang Y., Chen Y., Zheng J., Liu L., Zhang Q. (2022). Three-Dimensional Printing Self-Healing Dynamic/Photocrosslinking Gelatin-Hyaluronic Acid Double-Network Hydrogel for Tissue Engineering. ACS Omega.

[46] Grandjean T., Perumal N., Manicam C., Matthey B., Wu T., Thiem D.G.E., Stein S., Henrich D., Kämmerer P.W., Al-Nawas B. (2024). Towards optimized tissue regeneration: a new 3D printable bioink of alginate/cellulose hydrogel loaded with thrombocyte concentrate. Front. Bioeng. Biotechnol..

[47] Guimarães C.F., Gasperini L., Marques A.P., Reis R.L. (2020). The stiffness of living tissues and its implications for tissue engineering. Nat. Rev. Mater..

[48] Agache P.G., Monneur C., Leveque J.L., De Rigal J. (1980). Mechanical Properties and Young’s Modulus of Human Skin in Vivo. Arch. Dermatol. Res..

[49] Wang Y., Wang G., Luo X., Qiu J., Tang C. (2012). Substrate stiffness regulates the proliferation, migration, and differentiation of epidermal cells. Burns.

[50] Engler A.J., Sen S., Sweeney H.L., Discher D.E. (2006). Matrix Elasticity Directs Stem Cell Lineage Specification. Cell.

[51] Nakagawa S., Pawelek P., Grinnell’ F. (1989). Extracellular Matrix Organization Modulates Fibroblast Growth and Growth Factor Responsiveness. Exp. Cell Res..

[52] Skopinska-Wisniewska J., Tuszynska M., Kaźmierski Ł., Bartniak M., Bajek A. (2024). Gelatin–Sodium Alginate Hydrogels Cross-Linked by Squaric Acid and Dialdehyde Starch as a Potential Bio-Ink. Polymers.

[53] dos Santos Araújo P., Belini G.B., Mambrini G.P., Yamaji F.M., Waldman W.R. (2019). Thermal degradation of calcium and sodium alginate: A greener synthesis towards calcium oxide micro/nanoparticles. Int. J. Biol. Macromol..

[54] Nikolova M.P., Chavali M.S. (2019). Recent advances in biomaterials for 3D scaffolds: A review. Bioact. Mater..

[55] Correia D.M., Padrão J., Rodrigues L.R., Dourado F., Lanceros-Méndez S., Sencadas V. (2013). Thermal and hydrolytic degradation of electrospun fish gelatin membranes. Polym. Test..

[56] Dai Z., Ronholm J., Tian Y., Sethi B., Cao X. (2016). Sterilization techniques for biodegradable scaffolds in tissue engineering applications. J. Tissue Eng..

[57] Mukasheva F., Adilova L., Dyussenbinov A., Yernaimanova B., Abilev M., Akilbekova D. (2024). Optimizing scaffold pore size for tissue engineering: insights across various tissue types. Front. Bioeng. Biotechnol..

[58] Bartoš M., Suchý T., Foltán R. (2018). Note on the use of different approaches to determine the pore sizes of tissue engineering scaffolds: What do we measure?. Biomed. Eng. Online.

[59] Kilic Bektas C., Kimiz I., Sendemir A., Hasirci V., Hasirci N. (2018). A bilayer scaffold prepared from collagen and carboxymethyl cellulose for skin tissue engineering applications. J. Biomater. Sci. Polym. Ed..

[60] Ramasamy S., Davoodi P., Vijayavenkataraman S., Teoh J.H., Thamizhchelvan A.M., Robinson K.S., Wu B., Fuh J.Y.H., DiColandrea T., Zhao H. (2021). Optimized construction of a full thickness human skin equivalent using 3D bioprinting and a PCL/collagen dermal scaffold. Bioprinting.

[61] Heimbuck A.M., Priddy-Arrington T.R., Sawyer B.J., Caldorera-Moore M.E. (2019). Effects of post-processing methods on chitosan-genipin hydrogel properties. Mater. Sci. Eng., C.

[62] Krizanova O., Penesova A., Sokol J., Hokynkova A., Samadian A., Babula P. (2022). Signaling pathways in cutaneous wound healing. Front. Physiol..

[63] Orive G., Anitua E. (2021). Platelet-rich therapies as an emerging platform for regenerative medicine. Expert Opin. Biol. Ther..

[64] Li Y., Zhang Y., Wang Y., Yu K., Hu E., Lu F., Shang S., Xie R., Lan G. (2022). Regulating wound moisture for accelerated healing: A strategy for the continuous drainage of wound exudates by mimicking plant transpiration. Chem. Eng. J..

[65] Patel D.K., Jung E., Priya S., Won S.Y., Han S.S. (2024). Recent advances in biopolymer-based hydrogels and their potential biomedical applications. Carbohydr. Polym..

[66] Khan M.U.A., Stojanović G.M., Abdullah M.F.B., Dolatshahi-Pirouz A., Marei H.E., Ashammakhi N., Hasan A. (2024). Fundamental properties of smart hydrogels for tissue engineering applications: A review. Int. J. Biol. Macromol..

[67] Jensen P.J., Graham J.P., Busch T.K., Fitz O., Jayanadh S., Pashuck T.E., Gonzalez-Fernandez T. (2025). Biocompatible composite hydrogel with on-demand swelling-shrinking properties for 4D bioprinting. Biomater. Sci..

[68] Sharma D., Srivastava S., Kumar S., Sharma P.K., Hassani R., Dailah H.G., Khalid A., Mohan S. (2023). Biodegradable Electrospun Scaffolds as an Emerging Tool for Skin Wound Regeneration: A Comprehensive Review. Pharmaceuticals.

[69] Chen G., Tang W., Wang X., Zhao X., Chen C., Zhu Z. (2019). Applications of hydrogels with special physical properties in biomedicine. Polymers.

[70] Pan T., Song W., Cao X., Wang Y. (2016). 3D Bioplotting of Gelatin/Alginate Scaffolds for Tissue Engineering: Influence of Crosslinking Degree and Pore Architecture on Physicochemical Properties. J. Mater. Sci. Technol..

[71] Annor A.H., Tang M.E., Pui C.L., Ebersole G.C., Frisella M.M., Matthews B.D., Deeken C.R. (2012). Effect of enzymatic degradation on the mechanical properties of biological scaffold materials. Surg. Endosc..

[72] Guo S., DiPietro L.A. (2010). Factors affecting wound healing. J. Dent. Res..

[73] Dhivya S., Padma V.V., Santhini E. (2015). Wound dressings - A review. Bio.

[74] Díaz-Herrera M.Á., Martínez-Riera J.R., Verdú-Soriano J., Capillas-Pérez R.M., Pont-García C., Tenllado-Pérez S., Cunillera-Puértolas O., Berenguer-Pérez M., Gea-Caballero V. (2021). Multicentre study of chronic wounds point prevalence in primary health care in the southern metropolitan area of Barcelona. J. Clin. Med..

[75] Lukin I., Erezuma I., Garcia-Garcia P., Reyes R., Evora C., Kadumudi F.B., Dolatshahi-Pirouz A., Orive G. (2023). Sumecton reinforced gelatin-based scaffolds for cell-free bone regeneration. Int. J. Biol. Macromol..

[76] International Organization for Standardization (ISO) (2009) ISO 10993-5: Biological evaluation of medical devices – Part 5: Tests for in vitro cytotoxicity.

[77] Anitua E., Zalduendo M., Troya M., Erezuma I., Lukin I., Hernáez-Moya R., Orive G. (2022). Composite alginate-gelatin hydrogels incorporating PRGF enhance human dental pulp cell adhesion, chemotaxis and proliferation. Int. J. Pharm. X..

[78] Place E.S., Evans N.D., Stevens M.M. (2009). Complexity in biomaterials for tissue engineering. Nat. Mater..

[79] Zhang Y.S., Khademhosseini A. (2017). Advances in engineering hydrogels. Science.

[80] Ribeiro A., Pereira-Leite C., Rosado C., Aruci E., Colley H.E., Kortekaas Krohn I., Baldea I., Pantelić I., Fluhr J.W., Simões S.I. (2025). Enhancing Transcutaneous Drug Delivery: Advanced Perspectives on Skin Models. JID Innov..

[81] Zomer H.D., Trentin A.G. (2018). Skin wound healing in humans and mice: Challenges in translational research. J. Dermatol. Sci..

[82] Parnell L.K.S., Volk S.W. (2019). The Evolution of Animal Models in Wound Healing Research: 1993-2017. Adv. Wound Care.

[83] (2010) DIRECTIVE 2010/63/EU OF THE EUROPEAN PARLIAMENT AND OF THE COUNCIL of 22 September 2010 on the protection of animals used for scientific purposes (Text with EEA relevance).

[84] Frade M.A.C., Andrade T.A.M.d., Aguiar A.F.C.L., Guedes F.A., Leite M.N., Passos W.R., Coelho E.B., Das P.K. (2015). Prolonged viability of human organotypic skin explant in culture method (hOSEC). An. Bras. Dermatol..

[85] Min M., Egli C., Bartolome R., Sivamani R. (2024). Ex vivo Evaluation of a Liposome-Mediated Antioxidant Delivery System on Markers of Skin Photoaging and Skin Penetration. Clin. Cosmet. Invest. Dermatol..

[86] Mathew-Steiner S.S., Roy S., Sen C.K. (2021). Collagen in wound healing. Bioengineering.

[87] Smith M.M., Melrose J. (2015). Proteoglycans in Normal and Healing Skin. Adv. Wound Care.

[88] Puri S., Coulson-Thomas Y.M., Gesteira T.F., Coulson-Thomas V.J. (2020). Distribution and Function of Glycosaminoglycans and Proteoglycans in the Development, Homeostasis and Pathology of the Ocular Surface. Front. Cell Dev. Biol..

[89] Coenen A.M.J., Bernaerts K.V., Harings J.A.W., Jockenhoevel S., Ghazanfari S. (2018). Elastic materials for tissue engineering applications: Natural, synthetic, and hybrid polymers. Acta Biomater..

[90] Zhou M., Gomes M.P., Elgersma A., Korkmaz H.I., Boekema B.K.H.L., Groot M.L. (2025). Multiscale investigation of collagen structure in human skin and gel matrices using polarization resolved second harmonic generation microscopy. Sci. Rep..

[91] Cuño-Gómiz C., Tutusaus A., Savino F., Rider P., Colell A., Morales A., Marí M. (2026). Picrosirius red, polarized light microscopy, and curvelet transform analysis: An improved method for liver fibrosis assessment. Tissue Cell.

[92] Schneider C., Rasband W., Eliceiri K. (2012). NIH Image to ImageJ: 25 years of image analysis. Nat Methods.

[93] R Core Team (2023) R: A Language and Environment for Statistical Computing.

[94] Echave M.C., Pimenta-Lopes C., Pedraz J.L., Mehrali M., Dolatshahi-Pirouz A., Ventura F., Orive G. (2019). Enzymatic crosslinked gelatin 3D scaffolds for bone tissue engineering. Int. J. Pharm. X..

[95] Cannella V., Altomare R., Leonardi V., Russotto L., Di Bella S., Mira F., Guercio A. (2020). In Vitro Biocompatibility Evaluation of Nine Dermal Fillers on L929 Cell Line. Biomed Res. Int..

