# Supplementary material for: Ready-to-use 3D bioprinted scaffolds from natural materials loaded with patient’s PRGF for personalized skin regeneration
Source: iScience. 2026 Jun 8;29(6):116185. doi: 10.1016/j.isci.2026.116185 (PMC13264137; doi:10.1016/j.isci.2026.116185)

iScience, Volume *29*

Supplemental information

Ready-to-use 3D bioprinted scaffolds from natural materials loaded with patient's PRGF for personalized skin regeneration

Lidia Maeso, Eduardo Anitua, Roberto Tierno, Mohammad Hamdan Alkhraisat, Edurne Alonso, Jon Luzuriaga, Jon Zarate, Felipe Goñi, Tatiane Eufrásio-da-Silva, Mohammadsadegh Nadimifar, Sadegh Ghorbani, Aziz Maleki, Alireza Dolatshahi-Pirouz, and Gorka Orive

Supplemental Information

Table S1. Characteristic FTIR absorption bands of gelatin and alginate in the 3D bioprinted scaffolds.

Wavenumbers (cm^{-1})	Gelatin	Alginate
**3232**	OH;<br>NH, Amide A-Amide III	OH
**2917**	CH_2	CH_2
**2862**	CH	
**1598**	C=O; CN, Amide I	COO-
**1532**	C-N-H, Amide II	
**1403**		COO-
**1304**		C-O
**1228**	C-N; N-H, Amide III	
**1010**		C-O-C
**804**	OH	

Figure S1. Ex vivo skin explant study using Human Organotypic Skin Explant Cultures (hOSECs). Cell viability assessment, measuring metabolic activity (RES%) (a) and cell damage (LDH%) (b) of skin cells across three experimental groups: healthy skin (C+), burn injury (C–), burn & commercially available balsam product (Commercial product). (c) Inflammatory response, analyzing the effect of the burn condition on the synthesis of the pro-inflammatory cytokine IL-6 after 48 hours in the same experimental groups (C+, C–, Commercial product). Elastin (d) and collagen (e) quantification across the three experimental groups to assess extracellular matrix remodeling and tissue regeneration. Statistical significance is indicated as follows: not significant (ns), $p < 0.05$ (*), $p < 0.01$ (**), $p < 0.001$ (***), $p < 0.0001$ (****). See also Figure 5 and Figure 6.

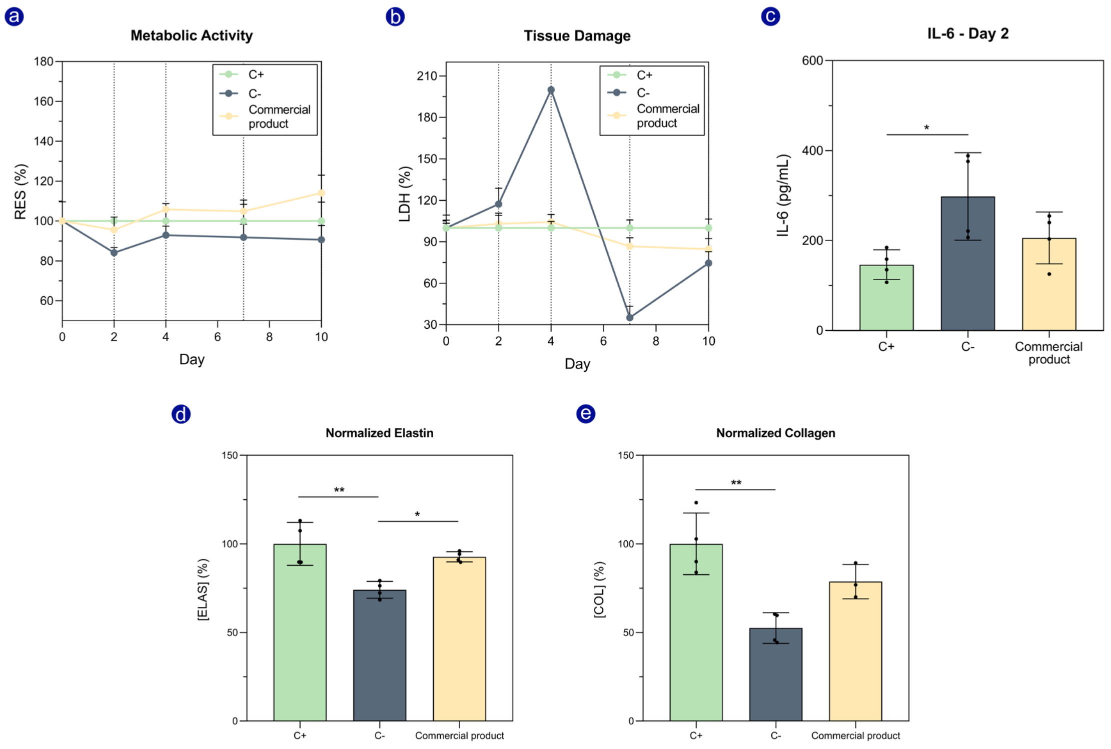

Figure S2. Photographs of the four alginate–gelatin bioink formulations prepared under different composition ratios. Visual differences in color and texture can be observed among the formulations.

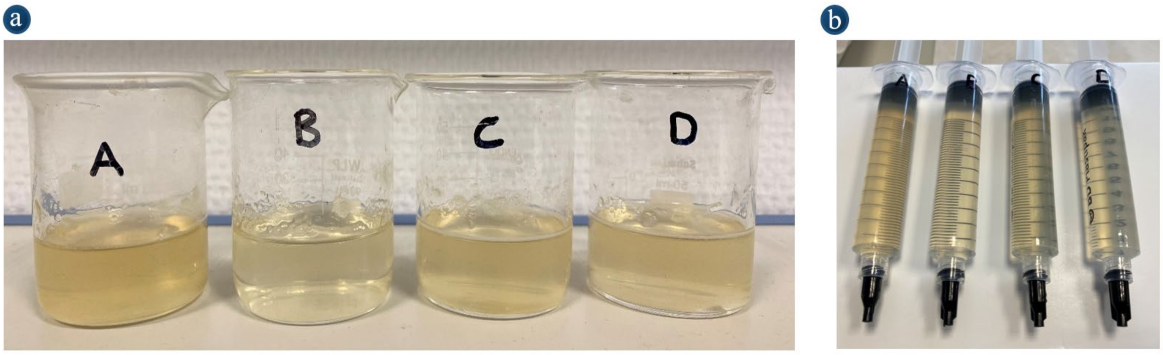

Figure S3. SEM images of bioprinted scaffolds after 24 h and 72 h of incubation in fibroblast culture medium (FM), with and without cells. Prior to incubation, scaffolds were rehydrated in PBS or PRGF (Scale bar = 10 μm).

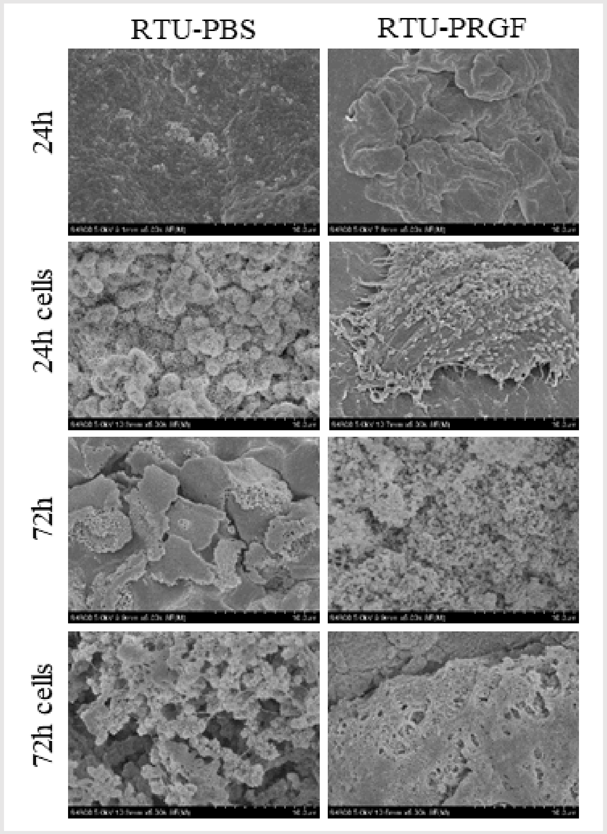

Supplement: Document S1. Figures S1–S3 and Table S1 [file mmc1.pdf]
